# Vocal Fold Fibroblasts in Reinke’s Edema Show Alterations Involved in Extracellular Matrix Production, Cytokine Response and Cell Cycle Control

**DOI:** 10.3390/biomedicines9070735

**Published:** 2021-06-26

**Authors:** Magdalena Grill, Isaac Lazzeri, Andrijana Kirsch, Nina Steurer, Tanja Grossmann, Michael Karbiener, Ellen Heitzer, Markus Gugatschka

**Affiliations:** 1 Division of Phoniatrics, Department of Otorhinolaryngology, Medical University of Graz, 8036 Graz, Austria; ma.grill@medunigraz.at (M.G.); nina.steurer@gmx.at (N.S.); tanja.grossmann@medunigraz.at (T.G.); michael@karbiener.at (M.K.); markus.gugatschka@medunigraz.at (M.G.); 2 Institute of Human Genetics, Diagnostic & Research Center for Molecular BioMedicine, Medical University of Graz, 8010 Graz, Austria; isaac.lazzeri@medunigraz.at (I.L.); ellen.heitzer@medunigraz.at (E.H.); 3 Global Pathogen Safety, Baxter AG, (part of Takeda), 1220 Vienna, Austria

**Keywords:** Reinke’s edema, RNA sequencing, vocal fold fibroblasts

## Abstract

The voice disorder Reinke’s edema (RE) is a smoking- and voice-abuse associated benign lesion of the vocal folds, defined by an edema of the Reinke’s space, accompanied by pathological microvasculature changes and immune cell infiltration. Vocal fold fibroblasts (VFF) are the main cell type of the lamina propria and play a key role in the disease progression. Current therapy is restricted to symptomatic treatment. Hence, there is an urgent need for a better understanding of the molecular causes of the disease. In the present study, we investigated differential expression profiles of RE and control VFF by means of RNA sequencing. In addition, fast gene set enrichment analysis (FGSEA) was performed in order to obtain involved biological processes, mRNA and protein levels of targets of interest were further evaluated. We identified 74 differentially regulated genes in total, 19 of which were upregulated and 55 downregulated. Differential expression analysis and FGSEA revealed upregulated genes and pathways involved in extracellular matrix (ECM) remodeling, inflammation and fibrosis. Downregulated genes and pathways were involved in ECM degradation, cell cycle control and proliferation. The current study addressed for the first time a direct comparison of VFF from RE to control and evaluated immediate functional consequences.

## 1. Introduction

Voice dysfunctions (dysphonia) reduce the quality of life of affected patients [1], and have economic consequences in professions with heavy vocal load [2]. Reinke’s edema (RE), a common, bilateral and benign lesion of the vocal folds (VF) is characterized by a swelling in the superficial lamina propria (LP), known as Reinke’s space [3]. The disease occurs almost exclusively in chronic smokers older than 40 years, predominantly women, with voice abuse as a trigger [4]. Patients affected with RE present with different levels of dysphonia, a hoarse, non-sustainable voice, and some also report breathing difficulties, depending on the lesion size [5]. Although a relation between smoking and voice abuse has been suggested [6,7], the initiating trauma leading to RE is not known. Continuous exposure to mechanical and chemical stressors, combined with the proliferation of micro-vessels, is proposed to lead to a damage of the capillary endothelium with subsequent extravasation, resulting in edema [8]. Even after surgery and cessation of noxious stimuli, phenotypical alterations are not completely reversible [9]. The development of RE appears to be a unique tissue phenotype and differs significantly from inflammatory processes of tissues several centimeters caudal, such as the tracheal mucosa [7]. Immuno-histologically, RE is characterized by a chaotic distribution of short and scattered connective fibers, a locally thickened and hyperplastic epithelial layer, thickening of the basement membrane, and edematous lakes [4,8]. The blood vessels tend to form pathological networks, are typically thin-walled and dilated [10]. The LP of RE specimens contains interstitial and inflammatory cells (mainly mastocytes and macrophages) [8]. Vocal fold fibroblasts (VFF), the main cell type of the LP, display a unique expression profile compared to fibroblasts from other tissues [11], and are considered to have an important role in mediating this disease [7,12]. Fibroblasts are primarily responsible for extracellular matrix (ECM) production, maintenance, and repair. Further, their ability to produce and respond to growth factors, chemokines, and cytokines allows reciprocal paracrine interactions, which influence the homeostasis of adjacent cell types, such as epithelial and endothelial cells [13]. 

As current treatment strategies for RE are only symptomatic, there is an urgent need for a better understanding of the underlying molecular causes of the disease. To date, RE VF tissue has been compared to VF polyps by microarray [14]. However, there is no comparison of RE VF or VFF to healthy controls available.

The aim of our study was to identify altered pathways in VFF isolated from RE tissue and to investigate functional consequences of these changes. We hypothesized that due to long-term irritants and noxious agents such as smoking and heavy voice use, gene expression in RE VFF is persistently altered, probably epigenetically regulated, and can be detected in primary cell cultures of VFF. Thus, to gain a better understanding of persistent changes in VFF of RE patients and further explore the molecular characteristics of the disease, we investigated the transcriptome of RE VFF compared to control samples.

## 2. Materials and Methods

### 2.1. Human Tissue Samples 

VF tissue samples from patients with confirmed RE (*n* = 18, mean age ± SD = 55.4 ± 10.1, all females) and healthy VF controls (*n* = 9, mean age ± SD = 59.4 ± 15.2, all females) were included in the study. Tissue samples were collected during phonosurgery (ENT University Hospital, Medical University of Graz, Graz, Austria), or received postmortem (Diagnostic and Research Institute of Pathology, Medical University of Graz). Procedures were approved by the local ethics committee of the Medical University of Graz (protocol numbers: 27-396 ex 14/15, approved 22 July 2015 and 29-036 ex 16/17, approved 07 October 2016). Where required, all participants provided written, informed consent. Samples and data used in this study were irreversibly anonymized. Diagnosis of RE was established by standard clinical, laryngoscopic and histological criteria. Additional patient information is listed in Supplementary Table S1.

### 2.2. Cell Culture

Sample processing was performed as described previously [12]. Cryostocks were thawed and cultivated in standard medium (SM), consisting of DMEM (4.5 g/L glucose, Sigma Aldrich, St. Louis, MO, USA), 10% fetal bovine serum (FBS) (Biowest, Nuaillé, France) and 100 µg/mL Normocin (Invivogen, San Diego, CA, USA) as previously described [15]. For experiments, cells with passages between 5 and 10 were used (passage No. mean ± SD for RE: 6.1 ± 0.87; for controls: 6.6 ±1.59), and cells were seeded at a density of 15,000 cells/cm^2^ and cultured for 72 h.

HUVEC cells (Lonza, Basel, Switzerland) were grown in Vasculife^®^ EnGS Endothelial Medium (Lifeline^®^ Cell Technology, Frederick, MD, USA) under standard cell culture conditions. A confluent T-25 cell culture flask (passage 3) was used for RNA isolation.

### 2.3. Proliferation Assay

CyQUANT cell proliferation assay kit (Invitrogen, Thermo Fisher Scientific, Waltham, MA, USA) was performed according to the manufacturer’s protocol. Briefly, cells were seeded at a density of 4800 cells in 0.2 mL SM/ well of a 96-well plate (Costar 3603, Sigma Aldrich, St. Louis, MO, USA) and grown for 72 h. Thereafter, cells were washed, images were taken and cells were frozen and stored at −80 °C until further analysis. Cells were seeded and measured in technical duplicates. For standard curve, 1 mL of a cell suspension of 200,000 cells/ mL was centrifuged (170× *g*, 5 min), washed, centrifuged again and the pellet was snap-frozen and stored at −80 °C until further analysis, according to the manufacturer’s manual.

### 2.4. Sample Collection

#### 2.4.1. Supernatant for Luminex^®^ Assays, ELISAs and Silver Stain

1 mL from 72 h cultures was collected, centrifuged for 10 min, 300× *g* at 4 °C, decanted and 200 µL aliquots were stored at −80 °C until further analysis.

#### 2.4.2. RNA Isolation

RNA from VFFs was isolated using Qiazol (Qiagen, Hilden, Germany) and miRNeasy kit (Qiagen, Hilden, Germany). Briefly, T-25 cell culture flasks were transferred on ice; cells were washed with ice-cold PBS, harvested with 700 µL Qiazol, and isolated according to the manufacturer’s protocol. Total RNA was quantified for RT-qPCR using NanoDrop 2000c Spectrophotometer (Thermo Fisher Scientific, Waltham, MA, USA), for RNA sequencing using Qubit™ RNA BR Assay on a Qubit^®^ Fluorometer (Thermo Fisher Scientific, Waltham, MA, USA) and assessed for quality using Agilent 2100 Bioanalyzer (Agilent Technologies, Santa Clara, CA, USA).

The RNA quality was uniformly high, with RNA integrity number (RIN) values above 9.5 in 24/27 samples (21 samples RIN 10, 2 samples RIN 9.8, 1 sample RIN 9.6). 3/27 samples had a RIN below 8 (1 sample RIN 7.5, 2 samples RIN 5–6) and fragmentation conditions during library preparation were adjusted for these samples accordingly.

#### 2.4.3. Cell lysates for Protein Analysis

T-25 cell culture flasks were transferred on ice. Cells were washed twice with ice-cold PBS and harvested in freshly prepared protein extraction buffer (PEB) consisting of 1× RIPA buffer (5× stock; Cell Biolabs Inc, San Diego, CA, USA), supplemented with 1x concentration of HALT™ protease and phosphatase inhibitor cocktail (100× stock; Thermo Scientific, Waltham, MA, USA) and 0.5 M EDTA Solution (100× stock; Thermo Scientific, Waltham, MA, USA). Cells were scraped off the flasks on ice. Cell lysates were homogenized using an ultrasound sonicator (3× 10 s), centrifuged (10 min, 13,250× *g*, 4 °C) and aliquots were stored at −80 °C. Protein concentrations were determined using the Pierce™ BCA Protein Assay kit (Thermo Scientific, Waltham, MA, USA) according to manufacturer’s instructions.

### 2.5. Sample Analysis

#### 2.5.1. RNA-seq and Enrichment Analysis

Sequencing libraries were prepared using 500 ng RNA per sample and the TruSeq^®^ Stranded total RNA HMR kit (Illumina, Eindhoven, Netherlands) according to manufacturer’s recommendation. Libraries were quantified using qPCR, pooled equimolarly and sequenced on an Illumina NextSeq 550 in a 150 cycle paired-end run obtaining an average of 46.2 million (range 27.9−67.2) paired-end reads per sample. Of those, an average of 41.3 million (~90%) reads were mapped successfully to the hg19 reference genome using STAR [16] and Salmon [17] aligners through the Bcbio RNA-seq workflow (version 1.1.5) [18]. The sequencing raw datasets have been deposited at the European Genome-phenome Archive (EGA). Available online: http://www.ebi.ac.uk/ega/ (accessed on 23 June 2021) under the study accession number EGAS00001005130. Statistics concerning the alignment were retrieved using samtools stats [19] and are provided in the Supplementary data (RNA-seq MultiQC report). TruSeq and poly a adaptors were trimmed using Atropos (version 1.1.2) [20]. Differential expression analysis was carried out in R (version 4.0.3) [21] using DESeq2 (version 1.30.0) [22]. Data were imported directly from the Bcbio pipeline results using the package bcbio RNA-seq (version 0.3.39) [23]. Unnormalized counts were used for differential expression analysis as suggested in the DESeq2 vignette. Genes having an absolute log2 fold change (FC) greater than 1 and an adjusted *p*-value (Benjamini–Hochberg) lower than 0.05 were selected, mitochondrial genes were removed.

Regularized log transformation (a DESeq2 function) was applied to the counts before plotting the heat map. The heat map was generated using the ComplexHeatmap package (version 2.6.2) [24]. Enrichment analyses were carried out using Fgsea package (version 1.16.0) [25]. Gene sets used for KEGG pathway, Gene Ontology and Hallmark enrichment analysis were downloaded from Gene Set Enrichment Analysis (GSEA). Available online: http://www.gsea-msigdb.org (accessed on 23 June 2021). Genes were sorted by adjusted *p* value, log2FC and highest expression levels and additionally evaluated for possible importance in RE formation and angiogenesis.

#### 2.5.2. Reverse Transcription (RT) and Quantitative Polymerase Chain Reaction (qPCR)

RNA was transcribed into cDNA using the QuantiTect RT kit (Qiagen, Hilden, Germany) including a genomic DNA elimination step, according to manufacturer’s protocol. qPCR was performed according to FAST cycling conditions from the Technical Manual for GoTaq^®^ qPCR Master Mix by Promega (version revised 12/18). For each sample, 10 ng cDNA, 200 nM of each primer (PrimePCR, Bio-Rad, Hercules, CA, USA) or self-designed primers from Microsynth (Balgach, Switzerland), listed in Table 1) and 5 µL GoTaq^®^-qPCR Mastermix containing BRYT Green^®^ Dye (Promega, Mannheim, Germany) were used in a final volume of 10 µL. For E2F1, 100 ng cDNA were used. cDNA and Mastermix were applied onto 384-well plates (FrameStar, Roche style; 4titude, Brooks Life Sciences, Manchester, UK) using an automated liquid handling workstation (Microlab STAR, Hamilton, Bonaduz, Switzerland). cDNA was amplified using the Bio-Rad CFX 384 real-time PCR Detection System (Bio-Rad, Hercules, CA, USA) and quantified on CFX Maestro 1.1 Software (version 4.1.2433.1219, Bio-Rad, Hercules, CA, USA). Self-designed primers were validated and melting curves were performed for all qPCR runs. For detection of the cDNA of one gene of interest, all samples were run on the same qPCR plate. Cq values of technical triplicates were averaged. The relative quantification of mRNAs of interest was calculated based on the 2^−ΔΔCq^ method [26] with minor adjustments: the geometric mean of Cq values of the reference RNAs HPRT and UXT were used as internal normalization factor, and one sample control was used as a reference to calculate ΔΔCq values.

#### 2.5.3. SDS-PAGE and Western Blot Analysis

25 µg protein per sample were mixed with Laemmli buffer (Bio-Rad, Hercules, CA, USA), reduced with DTT, denatured at 95 °C for 5 min and separated on Mini-Protean TGX or Criterion TGX Precast Gels, both 4−20% (Bio-Rad, Hercules, CA, USA) with TGS buffer (Bio-Rad, Hercules, CA, USA) at 175 V for 1h. Proteins were transferred onto Nitrocellulose membrane, 0.2 µm pore size (Bio-Rad, Hercules, CA, USA) with 0.5 A for 90 min at 4 °C. Blots were blocked with either 5% BSA (US Biological, Salem, MA, USA) or 5% skim milk powder (Sigma Aldrich, St. Louis, MO, USA) in TBS buffer (Bio-Rad, Hercules, CA, USA) containing 0.1% Tween 20 (Sigma Aldrich, St. Louis, MO, USA) for 1h, incubated with primary antibodies overnight at 4 °C, and secondary antibodies for 1h at RT, each incubation followed by 5 × 5 min wash steps. Antibodies, blocking reagents and dilutions are listed in Supplementary Table S2. HRP signal was developed using SuperSignal West Pico Chemiluminescent Substrate (Thermo Scientific, Waltham, MA, USA) and detected using Bio-Rad Chemidoc Touch (Bio-Rad, Hercules, CA, USA). Densitometric analysis was conducted using ImageLab™ software (version 6.0.1, Bio-Rad, Hercules, CA, USA). Standard band intensity of each target was normalized to the corresponding GAPDH standard band intensity. The mean of these normalized sample values over all samples on one blot was used as blot specific normalization factor.

#### 2.5.4. Pepsin Digestion, SDS-PAGE and Silver Stain

Analysis of fibrillar collagens was performed as described previously [15]. Briefly, pepsin-digested supernatants and a standard (VitroCol^®^ human type I collagen solution, Advanced BioMatrix, San Diego, CA, USA) were applied to 3−8% Criterion XT Tris-Acetate gels (Bio-Rad, Hercules, CA, USA). After electrophoresis, the gel was fixed and stained using the SilverQuest™ Silver Staining kit (Thermo Scientific, Waltham, MA, USA) according to the manufacturer’s protocol. Gel images were acquired with the ChemiDoc Touch system (Bio-Rad, Hercules, CA, USA) and densitometric analysis was conducted using ImageLab™ software (version 6.0.1, Bio-Rad, Hercules, CA, USA). For each gel, the mean of the type I collagen standard band intensity was used for normalization.

#### 2.5.5. ELISA 

All assays were performed according to manufacturer’s instructions. Samples were diluted in provided dilution buffers, optimal dilution of samples were chosen from previous experiments or determined in a pretest. For GREM2 BioAssay™ ELISA Kit (human) (United States Biological, Salem, MA, USA), samples were diluted 1:2 and a standard curve was generated using 0.0625 to 4 ng/mL of GREM2 standard. Hyaluronan (HA) was measured using the Quantikine ELISA (R&D Systems, Abingdon, UK), samples were prediluted 1:120 and a calibration curve was generated using 0.625 to 40 ng/mL HA standard. For the RayBio^®^ Human TGM2 ELISA kit (RayBiotech, Norcross, GA, USA), samples were used undiluted and a TGM2 standard was used to generate a calibration curve from 0.819 to 200 ng/mL. For analysis, OD values of samples and standards were measured using the Spectramax Plus 384 Microplate Reader (Molecular Devices, San Jose, CA, USA) and OD values at 540 nm were subtracted from values at 450 nm. Concentrations of samples were determined from standard curve, corrected by blank value (SM) and multiplied by the corresponding dilution factor.

For the LEGEND MAX™ Free Active TGF-β1 ELISA kit (BioLegend, San Diego, CA), samples were applied undiluted. For detection of total TGF-β1, LEGEND MAX™ Total TGF-β1 ELISA Kit (BioLegend, San Diego, CA) was used and samples were prediluted 1:50. For each ELISA, a calibration curve was generated using 7.8 to 500 pg/mL TGF-β1 standard. The analysis was performed as described above with one minor change: OD values at 570 nm were subtracted from values at 450 nm.

#### 2.5.6. Magnetic Luminex^®^ Assay

Proteins of interest were determined in supernatants using custom Human Magnetic Luminex^®^ Assays (R&D biosystems) for analysis of COL1α1, MMP-1, MMP-2, TIMP-1, VEGFA, VEGFC, platelet-derived growth factor (PDGF) DD, IL-6Rα and IL-1R1 according to manufacturer’s instructions. Standard curves were generated from provided analyte standards in the ranges outlined in Supplementary Table S3. SM was used as a blank. Sample dilutions were determined from previous experiments and are listed in Supplementary Table S3. Assays were measured on the Bio-Plex 200 assay reader and concentrations were calculated using the Bio-Plex Manager Software, version 6.2 (both Bio-Rad, Hercules, CA, USA).

### 2.6. Statistical Analysis

Single outliers were identified using Graphpad quickcalcs Grubbs outlier test and removed from the analysis. Data were analyzed using GraphPad Prism 8.3.0. (San Diego, CA, USA) Normal distribution was tested using D’Agostino–Pearson omnibus test and variances were compared by F test employment. For group comparison, student’s *t*-test or corresponding alternatives were conducted. When samples were not normally distributed, Mann–Whitney rank test was performed. Student’s *t*-test with Welch’s correction was conducted, where variances differed significantly.

## 3. Results

### 3.1. RNA-Seq Reveals a Total of 74 Differentially Expressed Genes in RE VFF Cells

**Figure 1:**
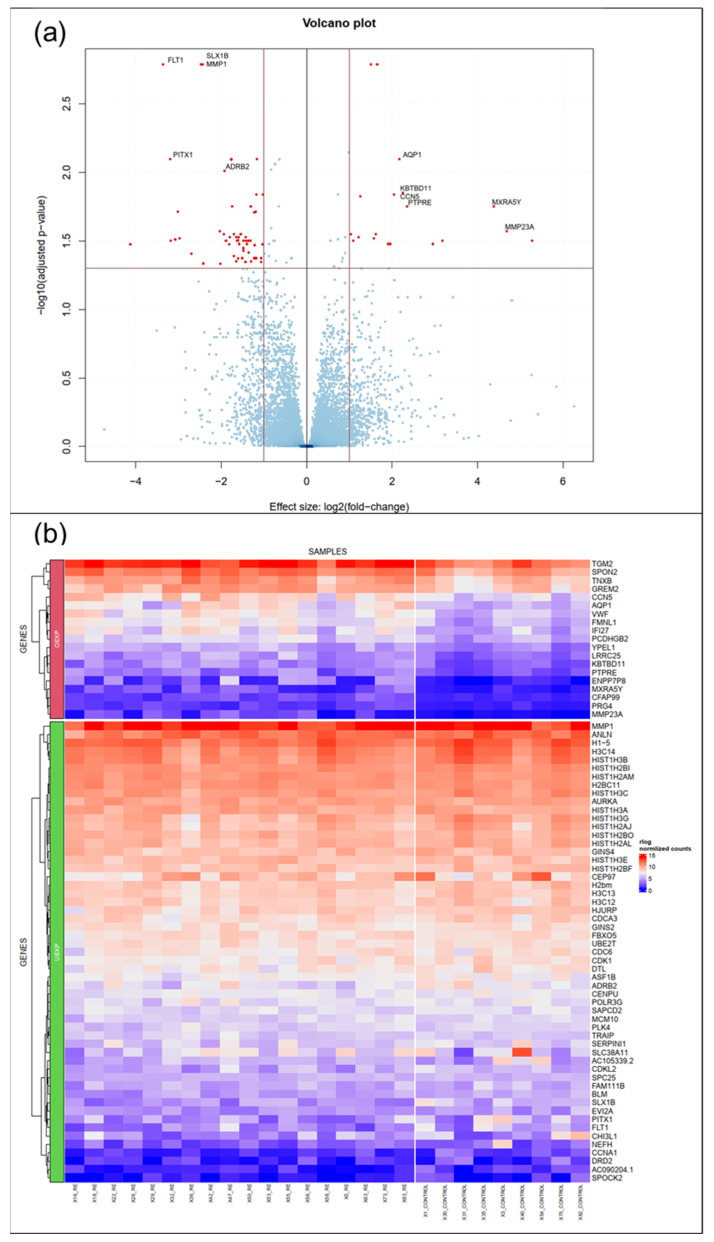
RNA-seq/ DESeq2 analysis revealed 74 differentially regulated genes. (**a**) Volcano plot summarizing the expression profile of healthy(control) VFF vs RE VFF (**b**) Heat map showing significantly upregulated (upper panel, overexpressed OEXP)/ downregulated genes (lower panel, underexpressed UEXP) with a log2FC above 1/ below −1 and an adjusted *p*-value below 0.05, further reduced by mitochondrial genes. Controls: *n* = 9, RE: *n* = 18.

A total of 27 VFF cultures from 18 RE patients and 9 controls were established and propagated. Since the generation of replicates was neither possible for individual patients nor the controls we used, we treated individual samples as biological replicates. Principle component analysis (PCA) of RNA-seq data did not reveal a clear clustering of RE VFF and controls (Supplementary data, Figure S1). However, DESeq2 analysis revealed 74 differentially regulated genes, 19 of them upregulated, 55 downregulated (Figure 1a,b, and Supplementary Tables S4 and S5). Of those, the top 7 upregulated genes, i.e., Aquaporin 1 (*AQP1*), von Willebrand Factor (*VWF*), Tenascin XB (*TNXB*), Transglutaminase 2 (*TGM2*), Cellular Communication Network Protein 5 (*CCN5*), Gremlin 2 (*GREM2*) and Spondin 2 (*SPON2*) and 3 downregulated genes, i.e., Matrix Metalloproteinase 1 (*MMP1*), Aurora kinase A (*AURKA*) and Ubiquitin-conjugating Enzyme E2T (*UBE2T*) were selected for further investigations and validation using qPCR.

### 3.2. Gene Expression of AQP1, and VWF, But Not Other Endothelial Cell Markers, Is Significantly Upregulated in RE VFF

**Figure 2:**
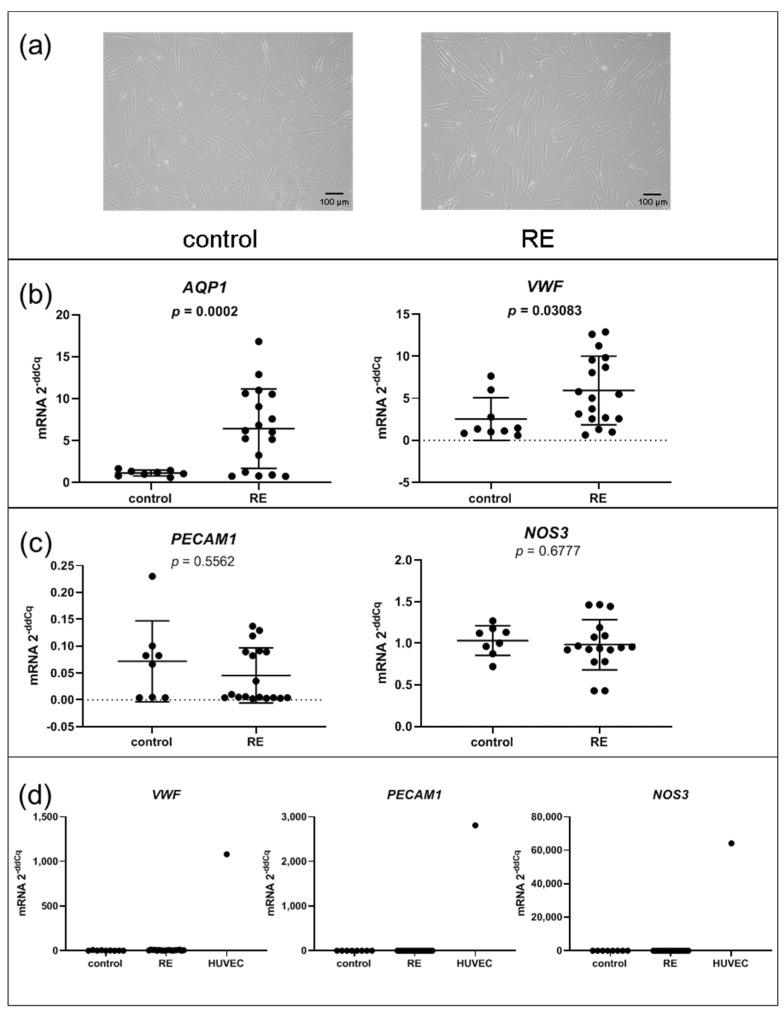
Gene expression of *AQP1*, and *VWF*, but not other endothelial cell markers, is upregulated in RE VFF. (**a**) morphology, (**b**) qPCR of *AQP1* and *VWF*, (**c**) qPCR of endothelial cell markers, (**d**) VFF compared to endothelial cell line (HUVEC). Graphs (**b**) and (**c**) show mean ± SD of 8 to 18 VFF samples per group (control: *n* = 8–9, RE: *n* = 17–18); groups were compared by Student‘s *t*-test, with Welch‘s correction if variances differed; in case of non-Gaussian distribution, Mann–Whitney rank test was conducted. *p* ≤ 0.05 was considered significant. Graph (**d**) shows single values of control VFF (*n* = 8–9), RE VFF (*n* = 17–18) or HUVEC (*n* = 1) to depict representative expression levels of these cell types.

Since AQP1 and VWF are predominantly expressed in endothelial cells, we wanted to rule out endothelial contamination in our cultivated cells. To this end, we examined the cell morphology, which for all fibroblast cell lines appeared in an elongated, spindle-like formation indicating these cells are indeed fibroblast cells (Figure 2a). Next, we confirmed upregulation of *AQP1* and *VWF* by qPCR. Both had significantly higher expression levels in RE VFF compared to controls (*p* = 0.002 and *p* = 0.0308) (Figure 2b). However, when assessing two additional endothelial cell markers, platelet and endothelial cell adhesion molecule (*PECAM1*) and endothelial nitric oxide synthase (*NOS3*) expression levels, we observed an equally low expression in RE VFF and control cells (Figure 2c). Compared to the human umbilical vein endothelial cell line (HUVEC), the *VWF* expression level was substantially lower in both, RE VFF and controls. Likewise, we observed a considerable overexpression of *PECAM1* and *NOS3* in HUVEC compared to our fibroblast cell lines (Figure 2d). Due to the use of only one representative, pooled donor sample of HUVEC, no statistical evaluation was performed. To further confirm the fibroblast lineage, we assessed expression levels of additional cell-type-specific markers in the RNA-seq dataset. While the fibroblast marker Vimentin (*VIM*) was highly abundant in both, RE VFF and controls, the epithelial cell marker E-cadherin (*CDH1*), and the muscle cell marker alpha-actinin 3 (*ACTN3*), were only detected in traces with median transcripts per million (TPM) below 0.05 (Supplementary Table S6).

### 3.3. Expression of Genes Associated with the ECM Composition, Some of Which Involved in the Interaction with TGF-β Family Members, Are Significantly Upregulated in RE VFF

**Figure 3:**
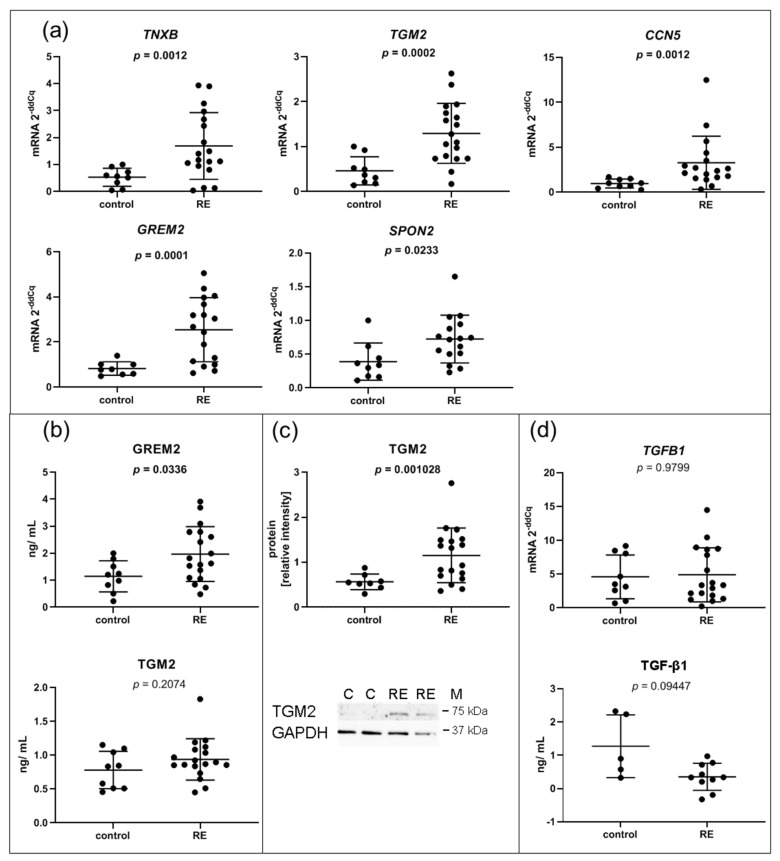
ECM related I: VFF from RE patients have increased expression levels of genes encoding for proteins related to the ECM and TGF-β family signaling. (**a**) Selected RNA-seq/DESeq2 differentially upregulated mRNAs compared to control samples were confirmed by qPCR, and (**b**) corresponding protein was measured in addition in the supernatant by Luminex and (**c**) in cell lysates by Western blotting; (**d**) *TGFB1* gene expression was measured by qPCR and total TGF-β1 protein in the supernatant was analyzed by ELISA. Graphs show mean ± SD of 8 to 18 samples per group (control: *n* = 8–9, RE: *n*= 17–18); total TGF-β1 was measured in 5–9 samples per group (control: *n* = 5, RE: *n* = 9); groups were compared by Student‘s *t*-test, with Welch‘s correction if variances differed; in case of non-Gaussian distribution, Mann–Whitney rank test was conducted. *p* ≤ 0.05 was considered significant. C = control, RE = Reinke‘s edema, M = marker.

Since an altered ECM composition is a hallmark of RE, it was not surprising that, compared to controls, several genes involved in the ECM composition were significantly upregulated in VFF cells. Upregulation of *TNXB*, *TGM2*, *CCN5*, *GREM2* and *SPON2*, all secreted proteins and components of the ECM, was confirmed by qPCR (Figure 3a). To corroborate these data at the protein level, we performed ELISA of three selected genes. While GREM2 was significantly upregulated in the 72 h supernatant of RE VFF cultures compared to control cells (Figure 3b), no significant difference for TGM2 was observed. CCN5 was out of range and could not be detected. However, Western blot analysis revealed an increase of TGM2 protein in cell lysates from RE VFF indicating an intracellular or cell surface localization (Figure 3c). Since TGM2 is involved in the deposition of the latent TGF-β complex to the cell surface, we tested if its altered expression had an impact on the availability of free or total TGF-β1 in the supernatant. Free TGF-β1 levels were below the detection limit of the ELISA kit. Nevertheless, total TGF-β1 was determined in a subset of samples. Though not significant, a clear trend towards lower levels of total TGF-β1 was visible (Figure 3d).

### 3.4. MMP1 Is Decreased in RE VFF Leading to Accumulation of COL1α2

**Figure 4:**
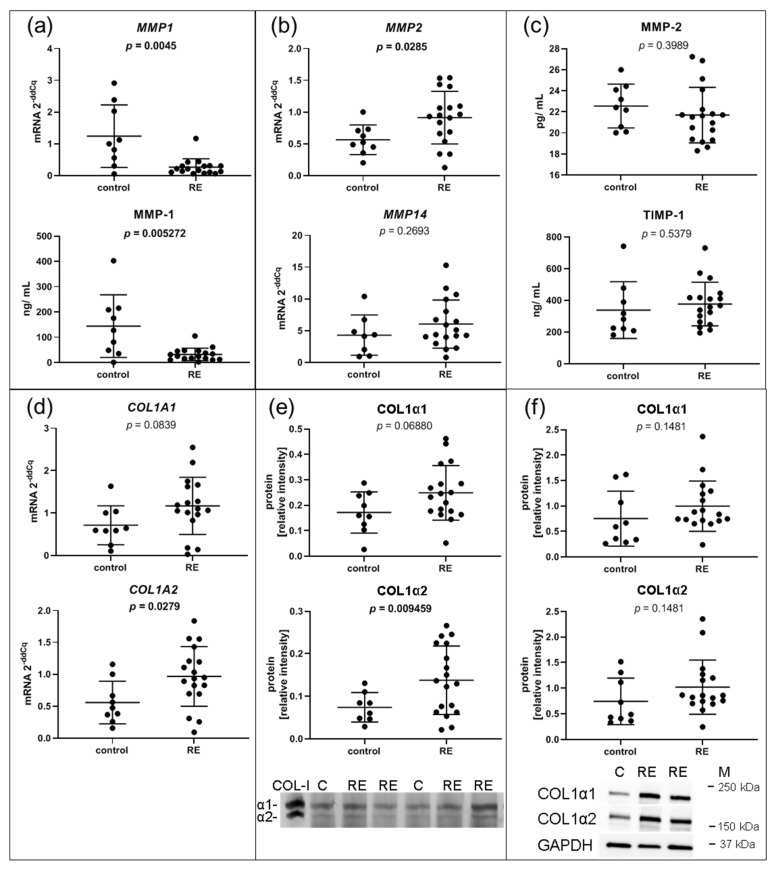
ECM related II: VFF from RE patients have decreased gene expression and protein levels of ECM degrading MMP1, and increased protein levels of its target COL1α2. (**a**) The selected RNA-seq/DESeq2 differentially downregulated mRNA of *MMP1* was confirmed by qPCR and corresponding protein in the supernatant by Luminex. (**b**) Gene expression of *MMP2* and *MMP14* that could compensate for the loss of MMP-1 was evaluated by qPCR. (**c**) Protein of MMP-2 and the MMP-1 inhibitor TIMP-1 was measured in the supernatant by Luminex. (**d**) Gene expression of MMP-1 targets *COL1A1* and *COL1A2* was measured by qPCR, as well as (**e**) on protein level in the supernatant by silver stain and (**f**) in the cell lysate by Western blotting. Graphs show mean ± SD of 8 to 18 samples per group (control: *n* = 8–9, RE: *n* = 17–18). Groups were compared by Student‘s *t*-test, with Welch‘s correction if variances differed; in case of non-Gaussian distribution, Mann–Whitney rank test was conducted. *p* ≤ 0.05 was considered significant. COL-I = Collagen 1 standard, C = control, RE = Reinke‘s edema, M = marker.

Since MMPs are responsible for the degradation of collagen and other proteins in the ECM, we further investigated MMP-1, the main collagen type I degrading enzyme. Although *MMP1* was highly abundant in both RE VFF and control cells, RNA-seq revealed a significant downregulation of *MMP1* in RE VFF, which was confirmed by qPCR (*p* = 0.0045) (Figure 4a). Moreover, decreased levels of extracellular MMP-1 protein were detected in the supernatant of RE VFF compared to controls (Figure 4a).

Gene expressions of *MMP14* and *MMP2* were additionally analyzed. While *MMP14* mRNA levels were not altered in RE VFF, *MMP2* mRNA was significantly increased (Figure 4b). Cell culture supernatant levels of MMP-2 protein, as well as the protein of tissue inhibitor of metalloproteinases 1 (TIMP-1), which inhibits MMP-1 action, were not significantly altered (Figure 4c). To delineate the impact of reduced MMP-1 levels, expression levels of MMP-1 targets, *COL1A1* and *COL1A2*, were evaluated. *COL1A1* was slightly, but not significantly upregulated on mRNA and protein level, respectively, both extra- and intracellularly (Figure 4d,e,f, respectively, upper panel). In contrast, *COL1A2* mRNA and extracellular COL1α2 protein were significantly increased; no significant changes were found on the intracellular protein level (Figure 4d,e,f, respectively, lower panel).

### 3.5. Pathways Involved in ECM Interactions and Turnover, Inflammation and Fibrosis Are Upregulated in RE, While Cell Proliferation Is Downregulated

**Figure 5:**
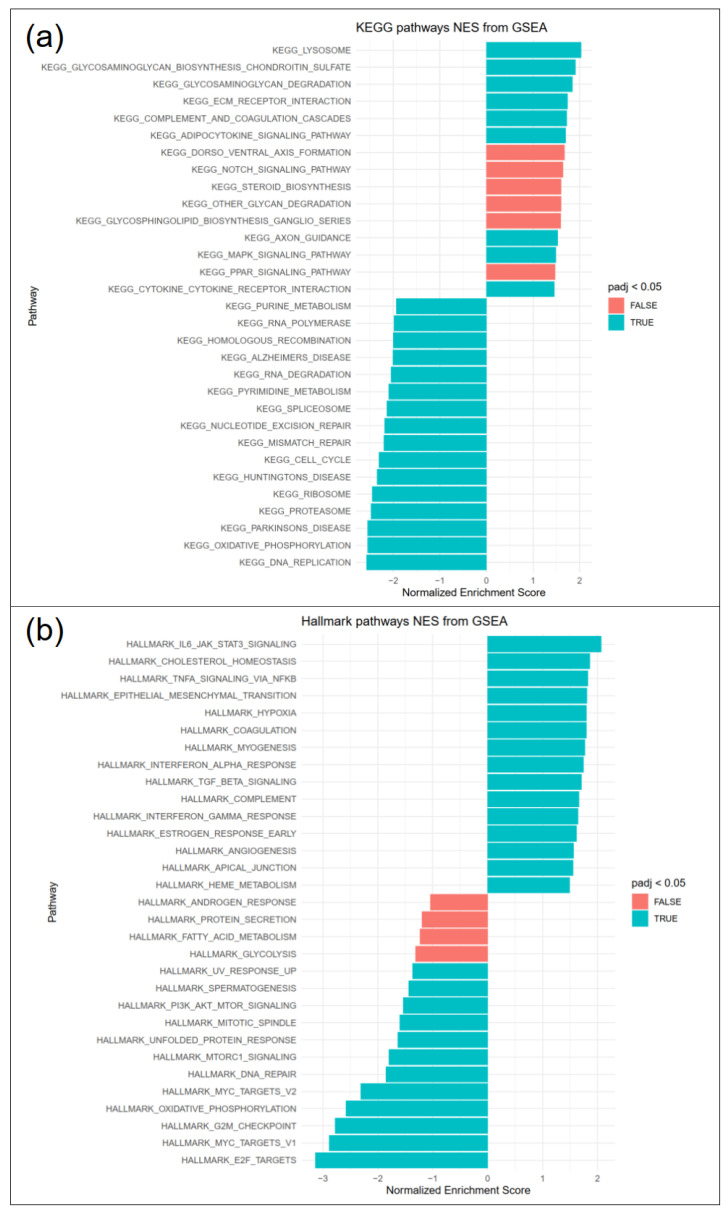
FGSEA pathway analysis revealed significantly regulated (**a**) KEGG and (**b**) hallmark pathways, 15 top and bottom regulated pathways are shown (blue padj < 0.05, red padj > 0.05).

Differentially expressed genes in RE VFF were subjected to fast gene set enrichment analysis (FGSEA). KEGG pathway analysis (Figure 5a) revealed upregulation of pathways involved in glycosaminoglycan turnover (lysosome, glycosaminoglycan biosynthesis chondroitin sulfate, glycosaminoglycan degradation) and ECM receptor interaction. Downregulated pathways included DNA replication, oxidative phosphorylation, proteasome, ribosome and cell cycle. Hallmark pathways (Figure 5b) included increased IL-6/JAK/STAT3 signaling, TNFα signaling via NFkB, epithelial mesenchymal transition, hypoxia, TGF-β signaling and decreased E2 factor (E2F) targets, myc targets, G2M checkpoint and oxidative phosphorylation, among others. Selected genes were additionally analyzed by qPCR.

### 3.6. RE VFFs Have Increased Levels of Inflammatory Cytokine Receptors IL1R1 and IL6R

**Figure 6:**
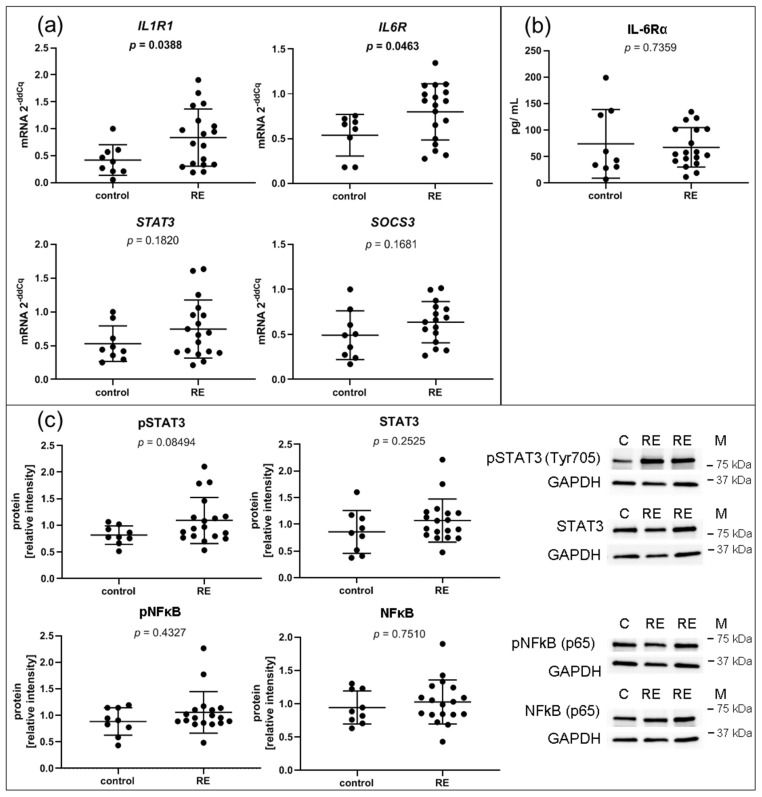
Inflammation: VFF from RE patients have increased gene expression levels of inflammatory cytokine receptors, but no intrinsically higher activation of IL1β/ NFkB or IL-6/ STAT3 pathways. (**a**) Gene expression of most regulated single components of significantly regulated hallmark pathway IL-6/JAK/STAT3 signaling, including *IL1R1*, *IL-6R*, *STAT3* and *SOCS3* were evaluated by qPCR; (**b**) presence of soluble IL-6Rα protein in the supernatant was measured by Luminex; (**c**) densitometric analysis of proteins by Western blots for pSTAT3 (Tyr705), STAT3, pNFkB (p65) and NFkB (p65); Graphs show mean ± SD of 8 to 18 samples per group (control: *n* = 8–9, RE: *n* = 18). Groups were compared by Student‘s *t*-test, with Welch‘s correction if variances differed; in case of non-Gaussian distribution, Mann–Whitney rank test was conducted. *p* ≤ 0.05 was considered significant. C = control, RE = Reinke‘s edema, M = marker.

To assess glycosaminoglycan turnover, *CSGALNACT2*, *GLB1* and *GALNS* were chosen for qPCR-based expression analysis but were not significantly deregulated (Supplementary Figure S2B). Likewise, the gene expressions of the transcription factor STAT3, a directly activated downstream target of the IL-6/JAK/STAT3 pathway, and SOCS3, negative feedback inhibitor of IL-6 signaling, respectively, were not significantly altered (Figure 6a). In contrast, other genes involved in the hallmark pathway IL6/JAK/STAT3 such as cytokine receptors *IL1R1* and *IL6R* were significantly overexpressed in RE VFF (Figure 6a). In the supernatant, IL-1R1 protein could not be detected, and IL-6Rα protein levels were not altered (Figure 6b). 

Intracellular/ cell surface protein of IL-1R1 and IL-6Rα could not be detected due to a lack of antibody specificity or low abundancy of receptors in the cells. However, IL-1β and IL-6 treatment of the cells confirmed an immediate response to these cytokines (unpublished observation). In addition, STAT3, NFκB and their activated phospho-proteins were measured by Western blot to investigate possible alterations in their signaling pathway activation (Figure 6c). Although not significant, p-STAT3 tended to be increased in RE samples, yet no changes were found in STAT3, p-NFκB or NFκB protein levels.

### 3.7. Cell Cycle, DNA Replication and E2F Targets Are Downregulated in RE VFF

**Figure 7:**
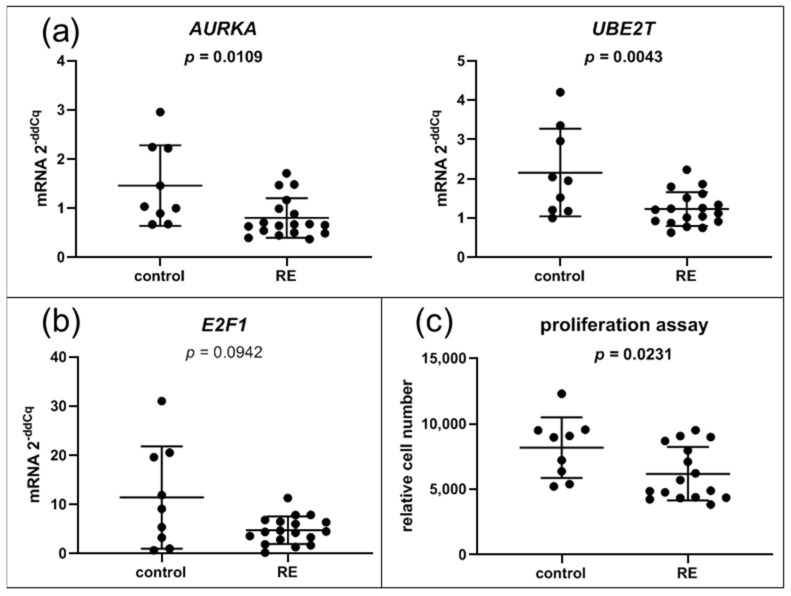
Cell cycle: VFF from RE patients have decreased expression levels of E2F target genes and decreased proliferation. (**a**) Gene expression of most altered single components of significantly regulated hallmark pathway E2F targets were evaluated by qPCR; (**b**) gene expression of the transcription factor *E2F1*, (**c**) proliferation assay. Graphs show mean ± SD of 9 to 18 samples per group (control: *n* = 9, RE: *n* = 16–18); Groups were compared by Student‘s *t*-test, with Welch‘s correction if variances differed; in case of non-Gaussian distribution, Mann–Whitney rank test was conducted. *p* ≤ 0.05 was considered significant.

Several downregulated gene sets like DNA replication, cell cycle and E2F targets overlap and share similar target genes. Excluding histons, one-third of the DESeq2 downregulated genes were associated with regulation via the E2F family. In addition, several histone genes that are only expressed during S phase of the cell cycle were also downregulated (Figure 1b). We were able to confirm downregulation of two E2F targets, *AURKA* and *UBE2T*, by qPCR (Figure 7a). The E2F transcription factor *E2F1* just missed significance in RNA-seq (log2FC = −1.08, *p*-value = 0.0007, padj = 0.087). Likewise, qPCR analysis showed a trend toward downregulation, but missed significance (*p* = 0.094) (Figure 7b). To confirm cell cycle downregulation, we performed proliferation assays. Indeed, proliferation of RE VFF was significantly reduced compared to control cells (*p* = 0.0231) (Figure 7c).

## 4. Discussion

Considerable knowledge of the influence of risk factors such as cigarette smoke and heavy voice use in RE has been gained from CSE-treatment and vibration experiments with immortalized cell lines and primary cells [7,12,27]. However, short-term treatments cannot completely represent the full extent of alterations observed after years of exposure to these irritant stimuli. Therefore, we isolated VFF from RE patients and healthy controls, compared their transcriptome by RNA-seq, verified alterations on important individual targets and determined functional consequences. Previously, it was reported that microarray expression analysis can differentiate between polyps and RE [14]. In our study, gene expression and enrichment analysis revealed a number of genes and signaling pathways which might be involved in the pathogenesis of RE.

### 4.1. Cultured RE VFF Reveal Alterations Associated with ECM Remodeling, Fibrosis and Angiogenesis

The ECM is a structural and regulatory network of extracellular components, mainly proteins and polysaccharides. Its tissue-specific composition has a major impact on physical properties such as viscoelasticity and vibration [28]. Thus, alterations in the homeostatic properties of the ECM can contribute to VF pathologies [11]. A prominent feature of RE pathology is the swelling in the superficial LP. We found that AQP-1, a water channel involved in edema formation and cell migration [29], was upregulated in RE VFF. Increased expression of AQP-1 was previously found in nasal polyp tissue, specifically in fibroblasts of the subepithelial area and the periphery of the seromucous glands, and in endothelial cells of venules [30]. Similar to RE, nasal polyp histology reveals edema formation. The exact localization of AQP-1-expressing fibroblasts has not been shown so far and can only be speculated. It is possible that some endothelial cells in RE samples have gone through an endothelial- to mesenchymal transition. During this transition, endothelial cells lose most of their endothelial markers and gain mesenchymal ones, leading to cytoskeletal alterations and changes in cell morphology [31]. This could explain the increased *VWF* expression found in RE samples, while other endothelial markers were not affected.

Differentially expressed genes in RE VFF, altering the ECM and further involved in wound healing and fibrosis, included *TGM2*, *MMP1*, *MMP2* and *COL1A2*. TGM2 has diverse functions depending on its (sub-/cellular) localization and cell type-specific expression [32]. Extracellularly/ on the cell surface, it cross-links itself and catalyzes cross-linking of other proteins, builds complexes with integrin and fibronectin, promotes cell adhesion and is increased during wound healing and tissue repair [32]. TGM2 interacts with several growth factor receptors [32], and is involved in the activation of latent TGF-β [33]. TGF-β has diverse roles in fibrosis, apoptosis, proliferation, differentiation, migration, adhesion, angiogenesis and ECM production. In endothelial cells, a functional relationship of TGF-β1 and TGM2 in angiogenesis was previously reported [34]. *TGM2* gene expression can be upregulated via TGF-β1 [34], NFkB [35], IL-6 [36], and downregulated via BMPs [37]. Neither TGF-β1, BMPs, p-NFkB (p65) nor p-STAT3 (Tyr705) were significantly changed in RE VFF compared to controls. Although not significant, total TGF-β1 tended to decrease in the supernatant of RE, which could indicate an increased deposition of latent TGF-β1 on the cell surface by TGM2. Basal expression of *TGM2* can further be epigenetically regulated via hypo-/hypermethylation of the proximal promoter [32], which needs to be investigated in future studies.

Interestingly, *MMP1* expression was significantly decreased in RE samples. As type I collagen is hydrolyzed by MMP-1 [38], decreased MMP-1 suggests an increase in type I collagen expression. Alternatively, if its inhibitor TIMP-1 is equally downregulated and the MMP-1/TIMP-1 ratio is preserved, this could compensate for the loss of MMP-1. We could observe a small but significant increase in COL1α2, however no significant increase in COL1α1 protein nor alterations in TIMP-1 protein levels. Thus, rather steady collagen levels have to be explained by a different mechanism. Gene expression of the membrane type I MMP (*MMP14*) with similar activity as MMP-1, [38] was, however, not altered. TGM2, upregulated in RE VFF, increases *MMP2* gene expression [39]. An increase in *MMP2* mRNA was found in RE samples, but no difference was seen on the protein level measured in the supernatant. CCN5, another important ECM protein upregulated in RE VFF, was previously shown to have a negative regulatory function on *COL1A1* [40]. Further, our medium did not contain additional L-ascorbic acid, an essential co-factor in the biosynthesis of collagen [41]. Therefore, it is possible that the ascorbic acid present in FBS was not sufficient for the collagen biosynthesis that would have been otherwise seen due to the reduced expression of MMP-1. Interestingly, *MMP3* was not significantly decreased in our RNA-seq data, despite an often, but not always coordinately regulated expression pattern with *MMP1* [42]. Therefore, it is possible that the *MMP1* downregulation in the RE samples is a consequence of epigenetic changes.

*TNXB*, upregulated in RE samples, is included in the ECM-receptor interaction pathway and regulates the production and assembly of collagen, the structure and stability of elastic fibers and organization of tissue structure [43]. Interestingly, TN-X expression is significantly downregulated during tumor progression in most cancers, and a high *TNXB* expression correlates with a good survival prognosis [44]. Likewise, a downregulation of *TGM2* was found in aggressive tumors and metastasis, while overexpression of *TGM2* led to reduction in tumor incidence and progression [45]. Thus, an upregulation of *TNXB* and *TGM2* in RE VFF goes in line with the benign nature of RE pathology, despite yearlong exposure to cigarette smoke and excessive voice abuse [46].

The ECM-associated protein CCN5 is regulated via the WNT pathway. It has important intra- and extracellular functions and is a key negative regulator of PPARy [47]. In fibroblasts, CCN5 has a negative regulatory role on fibrogenesis, interacts with and inhibits TGF-β-enhanced fibroblast viability and proliferation [40,48], inhibits the switch from fibroblasts to myofibroblasts and has a negative regulatory function on alpha-SMA [48]. This is in agreement with histological findings in RE specimens [49] and our current data, where no alpha-SMA expressing myofibroblasts were found. GREM2, also regulated via the WNT/β-catenin pathway, is a secreted protein that inhibits the activity of BMP 2 and 4 [50,51]. GREM2 and CCN5 both interact with or reduce the activity of BMP4 and therefore, prevent or reduce PPARy activation, respectively [47,50]. This might have implications in angiogenesis, as activation of PPARy in endothelial cells has predominantly antiangiogenic properties [52]. Although *CCN5* and *GREM2* gene expressions were increased, other classical upregulated targets of the WNT/β-catenin pathway were not altered or even downregulated (e.g., VEGF, Supplementary Figure S2A; downregulated hallmark pathway: c-myc, Figure 5b), indicating an alternative activation pathway or epigenetic regulation.

Taken together, we found transcriptional changes of several important players involved in ECM remodeling and altering ECM-receptor interactions, especially intervening with members of the TGF-β family. This could lead to an altered ECM composition as seen in RE, as well as a differential response to TGF-β family-mediated signaling processes and facilitate angiogenesis.

### 4.2. RE VFF Display Changes Leading to A Differential Inflammatory Response

Pathway analysis revealed an upregulation of the hallmark pathway IL-6/JAK/STAT3 signaling. Inflammatory cytokines, particularly IL-6, have previously been shown to elevate VEGF levels, induce angiogenesis and increase vascular permeability [53,54]. IL-6 can be induced via NFκB [55], which is activated by binding of the pro-inflammatory cytokine IL-1 to its receptor IL-1R1 [54]. IL-6 binds IL-6Rα either extracellularly as a soluble receptor or membrane bound on the cell surface [56]. Binding of this complex to the co-receptor gp130 leads to activation of further signaling cascades, such as STAT3, among others [57]. Via qPCR, we confirmed that *IL1R1* and *IL6R* mRNA levels were increased in RE compared to control samples. Beyond that, we found no indications from RNA-seq data that the expression of the corresponding cytokines themselves was increased. Considering that IL-1R1 and especially IL-6Rα can be shed or alternatively spliced, and an increased gene expression does not necessarily mean higher receptor levels on the cell surface [56], we evaluated the soluble forms of these receptors in the supernatant. While IL-1R1 was not detectable, IL-6Rα levels were not altered between RE and control samples. In addition, we confirmed that these cells do respond to IL-1β and IL-6 treatment. To evaluate this further, we investigated the activation of NFκB and STAT3 in RE VFF. Though p-STAT3 (Tyr705) levels tended to be higher, no significant changes were found in these pathways. This was especially interesting as it implies that these cells only have minimal alterations in intrinsic autocrine/paracrine signaling but could show a differential response to external stimuli. Our group previously showed an increased IL-6 release by VFF cells upon treatment with cigarette smoke extract [12]. In addition, the presence of macrophages and other inflammatory cells in RE tissue is evident [8]. To add to this context, we found an increased gene expression of SPON2, a cell adhesion protein of the ECM and pattern recognition protein essential in the initiation of the innate immune response [58]. A previous study showed that SPON2 promotes M1-like macrophage recruitment [59]. Thus, SPON2 could be involved in inflammatory infiltration seen in RE tissue specimens [60]. Increased GREM2 can, however, restrain the extent of inflammation via BMP2 inhibition [51]. Based on the current results, we propose an epigenetic alteration of the cytokine receptors *IL1R1* and *IL6R* in RE VFF, resulting in differential gene expression and possibly an altered response to cytokine stimulation by external stressors or infiltrating inflammatory cells. This could play a key role in edema formation and angiogenesis seen in RE, which remains to be investigated in the future.

### 4.3. RE VFF Show Cell Cycle Disruption

In a previous study, our group established a connection between an acute treatment with the RE risk factor cigarette smoke and cell cycle disruption [12]. The current study extends this finding and demonstrates a long-term impact on DNA replication, cell cycle disruption and reduced proliferation in RE VFF. Here, we exemplarily show that *AURKA*, encoding for a serin/threonine kinase strongly involved in mitosis [61], and *UBE2T*, involved in DNA repair [62], are both downregulated in RE VFF. These genes belong to the gene set of E2F targets and are regulated via E2F1. E2F1, the master regulator of cell cycle control, induces G1 to S transition and is crucial for cell cycle progression and proliferation, while decreased expression of E2F1 leads to cell cycle arrest [63]. *E2F1* itself is only expressed at very low levels and an increase is limited to a certain time point in the cell cycle. Therefore, missed significances in our study could be explained by a high variability of expression levels combined with a lack of sensitivity of the qPCR reaction with higher Cq values (~32 and above). Because a certain number of genes encoding for histones is tightly regulated and expressed only during the S phase of the cell cycle [64], the observed decrease in histone gene expression suggests a lower percentage of cells in the S phase. This is also reflected by the observed downregulation of cell cycle-related processes including E2F targets and decreased proliferation. Collectively, our previous study implied a negative regulatory role of the risk factor smoking on cell division, which is here shown to be further integrated into a long-lasting change in the VFF phenotype.

### 4.4. Study Limitations

RE tissue and consequently VFF from RE patients are very heterogeneous and therefore, no overall distinction between control and RE VFF was seen. Difficulties to receive healthy VF controls lead to a small number of controls obtained from deceased patients, meaning that there was no known pathology of the VF, but patients could have been treated for other pathologies, potentially influencing the healthiness of their VFF. To gather higher robustness of the data, much higher numbers of patient samples would be needed, which goes beyond financial limits and time constraints. Furthermore, we are aware that fibroblasts are only one cell type involved in this pathology, and future studies will be needed to explore the interaction of VFF with other cell types found in RE VF.

## 5. Conclusions

An altered expression of ECM components that contribute to ECM reorganization was found in RE VFF. In addition, increased levels of factors involved in TGF-β family signaling, as well as increased mRNA levels of receptors for inflammatory cytokines, suggest a differential response to cytokine stimulation in these cells. Transcriptional regulation towards cell cycle arrest, and therefore reduced proliferation could be a result of these alterations.

This is the first study to show alterations in VFF from RE compared to healthy controls on gene expression as well as protein level and depicts possible functional correlations and consequences. Future studies are needed to further analyze how these changes impact altered cellular interactions, differential cytokine response and resulting pathologies. Our RNA-seq dataset is a resource for the research community and can serve as a basis to build new hypotheses, gain new insights into molecular mechanisms, and lead to new approaches for treatment.

## Figures and Tables

**Table 1:** Primers used for qPCR.

Gene Name	Company	Product Number	Sequence Primer 1	Sequence Primer 2
*CCN5/WISP2*	Bio-Rad	qHsaCED0042203		
*GREM2*	Bio-Rad	qHsaCED0036866		
*TGM2*	Bio-Rad	qHsaCID0007428		
*AQP1*	Bio-Rad	qHsaCED0005827		
*SPON2*	Bio-Rad	qHsaCED0005641		
*TNXB*	Bio-Rad	qHsaCED0038143		
*IL1R1*	Bio-Rad	qHsaCID0010015		
*IL6R*	Bio-Rad	qHsaCED0045907		
*STAT3*	Bio-Rad	qHsaCID0010912		
*SOCS3*	Bio-Rad	qHsaCED0003543		
*AURKA*	Bio-Rad	qHsaCID0022123		
*UBE2T*	Bio-Rad	qHsaCID0011482		
*E2F1*	Bio-Rad	qHsaCID0016226		
*CSGALNACT2*	Bio-Rad	qHsaCID0016295		
*GLB1*	Bio-Rad	qHsaCID0012007		
*GALNS*	Bio-Rad	qHsaCID0007640		
*COL1A1*	Bio-Rad	qHsaCED0002181		
*TGFB1*	Microsynth		TACCTGAACCCGTGTTGCTC	GCTGAGGTATCGCCAGGAAT
*MMP1*	Microsynth		CACGCCAGATTTGCCAAGAG	GTTGTCCCGATGATCTCCCC
*MMP2*	Microsynth		ACCAAGAACTTCCGTCTGTCC	ATGTCAGGAGAGGCCCCATA
*MMP14*	Microsynth		CGGAGAATTTTGTGCTGCCC	AACCCTGACTCACCCCCATA
*COL1A2*	Microsynth		ACCACAGGGTGTTCAAGGTG	CAGGACCAGGGAGACCAAAC
*VWF*	Microsynth		CTCATCGCAGCAAAAGGAGC	ATGCTCATGCACTCCAGGTC
*PECAM1*	Microsynth		CGTCGAATACCAGTGTGTTGC	TCATCCACCGGGGCTATCA
*NOS3*	Microsynth		CGAGTGAAGGCGACAATCCT	ACAGGACCCGGGGATCAAAA

## Data Availability

Publicly available datasets were analyzed in this study. This data can be found at the European Genome-phenome Archive. Available online: http://www.ebi.ac.uk/ega/ EGAS00001005130 (accessed on 23 June 21).

## Supplementary Materials

The following are available online at https://www.mdpi.com/article/10.3390/biomedicines9070735/s1, Figure S1: Principal component analysis of RNA-seq, Figure S2: Targets of interest without significant alterations between RE VFF and controls, Table S1: Additional patient information, Table S2: Antibodies, blocking reagents and dilutions, Table S3: Standard curve ranges and sample dilutions used for Luminex, Table S4: List of differentially upregulated mRNAs detected by RNA-seq/DeSeq2, Table S5: List of differentially downregulated mRNAs detected by RNA-seq/DeSeq2, Table S6: List of cell type marker mRNAs detected by RNA-seq, RNA-seq MultiQC report.
