# Supplementary material for: Vocal Fold Fibroblasts in Reinke’s Edema Show Alterations Involved in Extracellular Matrix Production, Cytokine Response and Cell Cycle Control
Source: Biomedicines. 2021 Jun 26;9(7):735. doi: 10.3390/biomedicines9070735 (PMC8301432; doi:10.3390/biomedicines9070735)
Supplement: Supplementary file 1 [file biomedicines-09-00735-s001.zip › biomedicines-1222278-supplementary.pdf]

Supplementary data for the manuscript:

Vocal fold fibroblasts in Reinke's edema show alterations involved in extracellular matrix production, cytokine response and cell cycle control

Authors: Magdalena Grill, Isaac Lazzeri, Andrijana Kirsch, Nina Steurer, Tanja Grossmann, Michael Karbiener, Ellen Heitzer, Markus Gugatschka

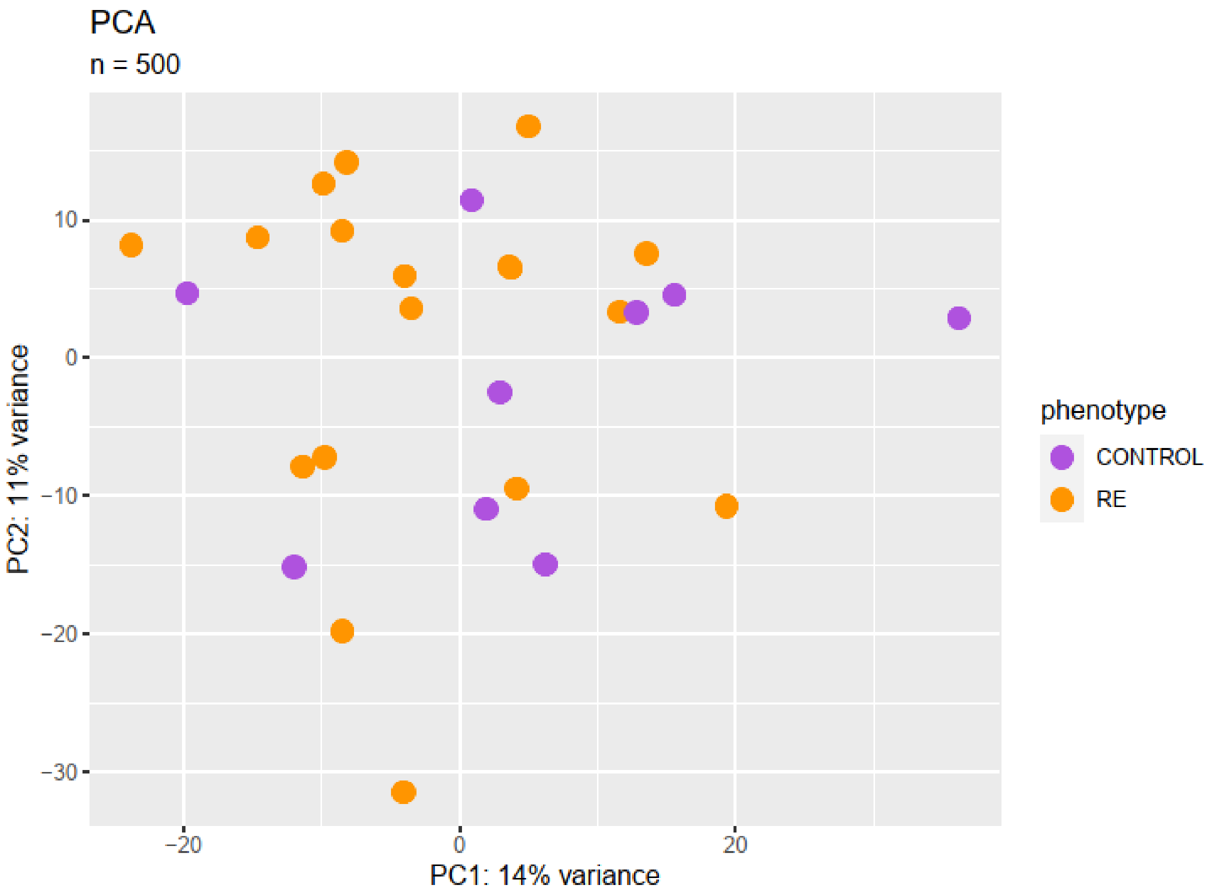

Figure S1: Principal component analysis of RNA-seq.

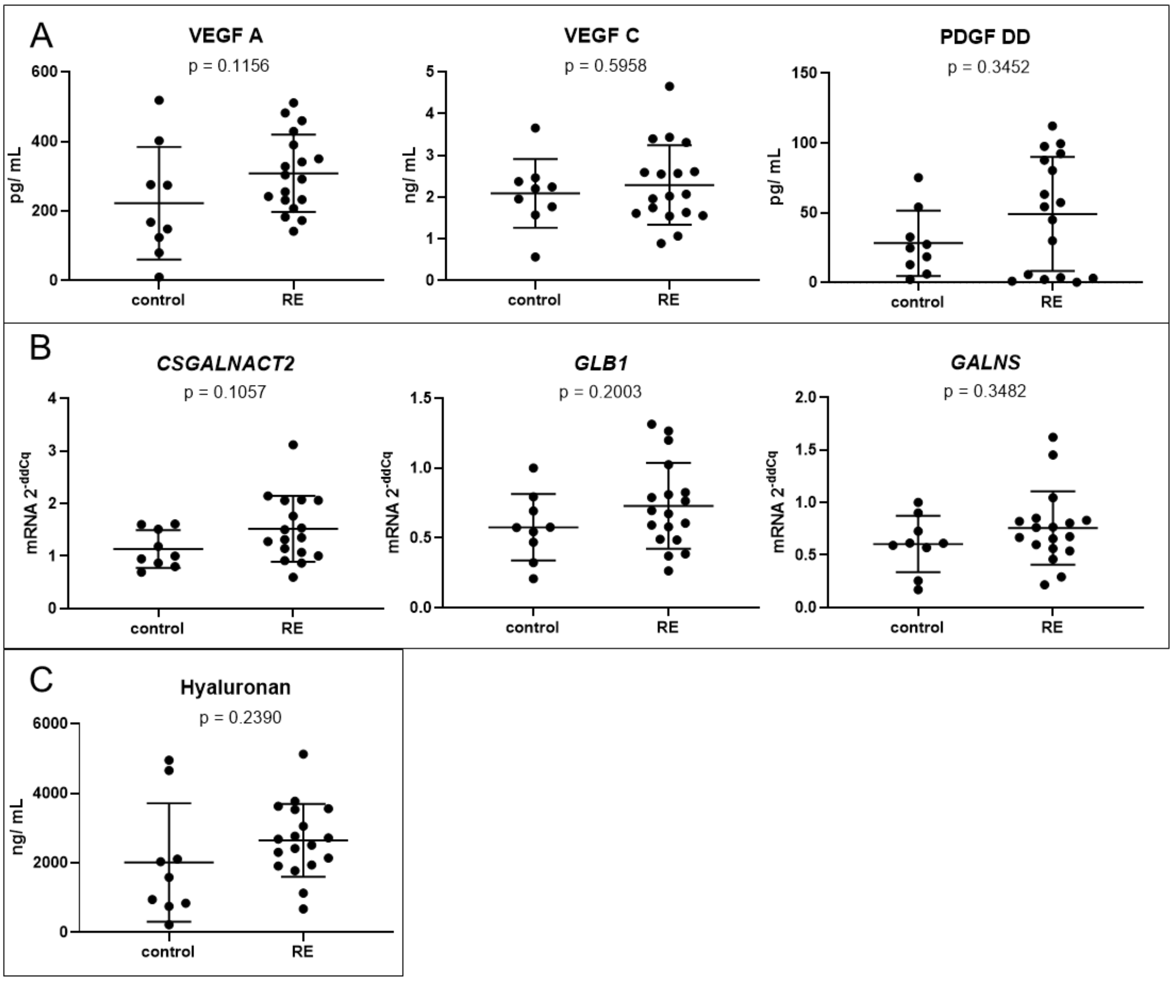

Figure S2: Targets of interest without significant alterations between RE VFF and controls. (A) Angiogenesis related, not significantly altered in the supernatant, detected on protein level by Luminex; (B) glycosaminoglycan synthesis/degradation related, not significantly regulated on mRNA level, detected by qPCR, (C) hyaluronan, not significantly altered, detected by ELISA. Graphs show mean ± SD of 9 to 18 samples per group (n=9-18); Groups were compared by student's t-test, with Welch's correction if variances differed; in case of non-Gaussian distribution, Mann Whitney rank test was conducted. p≤0.05 was considered significant.

Table S1: Additional patient information

sample name	VF pathology	age	sex
X1_CONTROL	-	76	female
X3_CONTROL	-	73	female
X30_CONTROL	-	49	female
X31_CONTROL	-	60	female
X35_CONTROL	-	63	female
X40_CONTROL	-	82	female
X54_CONTROL	-	45	female
X75_CONTROL	-	39	female
X82_CONTROL	-	48	female
X5_RE	RE	55	female
X16_RE	RE	31	female
X18_RE	RE	66	female
X22_RE	RE	58	female
X26_RE	RE	53	female
X29_RE	RE	66	female
X32_RE	RE	50	female
X36_RE	RE	72	female
X42_RE	RE	42	female
X47_RE	RE	51	female
X50_RE	RE	60	female
X53_RE	RE	56	female
X55_RE	RE	48	female
X56_RE	RE	71	female
X58_RE	RE	55	female
X63_RE	RE	48	female
X73_RE	RE	56	female
X83_RE	RE	60	female

Table S2: Antibodies, blocking reagents and dilutions

target	company	ProductNo	blocking and Ab solution	host		dilution	2nd Ab
**primary Ab**							
phospho-STAT3 (Tyr705)	CST	#9145	5% BSA	rabbit	mAb	1:2000	1:50 000
STAT3	CST	#12640	5% dry milk powder	rabbit	mAb	1:2000	1:50 000
phospho-NFκB (p65)	CST	#3033	5% BSA	rabbit	mAb	1:2000	1:50 000
NFκB (p65)	CST	#4764	5% BSA	rabbit	mAb	1:2000	1:50 000
TGM2	GeneTex	GTX111702	5% dry milk powder	rabbit	pAb	1:500	1:100 000
COL1A1	Nordic Bio Site	ABB-2670	5% dry milk powder	rabbit		1:2000	1:100 000
COL1A2	Proteintech Europe	14695-1-AP	5% dry milk powder	rabbit		1:2000	1:100 000
GAPDH	CST	#2118	5% dry milk powder	rabbit		1:5000	1:100 000
**secondary Ab**							
goat anti-rabbit IgG	abcam	ab6721	same as primary, either 5% BSA or 5% dry milk powder			1:50 000-1:100 000	

Table S3: Standard curve ranges and sample dilutions used for Luminex

protein	standard curve range	sample dilution factor
COL1A1	of 4 to 3000 pg/mL	50x
MMP1	15 to 10 700 pg/mL	50x
MMP2	300 to 80 000 pg/ mL	1x
TIMP1	18 to 13 000 pg/ mL	50x
VEGFA	7 to 2 000 pg/ mL	1x
VEGFC	15 to 12 500 pg/ mL	1x
PDGF DD	5 to 4 000 pg/mL	1x
IL6R	35 to 26 000 pg/ mL	1x
IL1R1	30 to 25 000 pg/ mL	1x

Table S4: List of differentially upregulated mRNAs detected by RNA-seq/DeSeq2

SYMBOL	ENSEMBL ID	log2FC	pvalue	padj	median_tpm_controls	median_tpm_re
TGM2	ENSG00000198959	1,5059	3,28E-07	0,002	19,313373	89,4714505
SPON2	ENSG00000159674	1,0391	4,30E-05	0,028	33,744713	73,165057
GREM2	ENSG00000180875	1,221	5,69E-05	0,03	3,358457	8,8534175
IFI27	ENSG00000165949	1,9086	0,000104	0,033	1,59003	7,316036
CCN5	ENSG00000064205	2,0491	1,14E-05	0,015	1,806938	5,5874145
TNXB	ENSG00000168477	1,2599	1,40E-05	0,015	2,070744	4,3631345
AQP1	ENSG00000240583	2,174	3,14E-06	0,008	0,492645	2,1685205
FMNL1	ENSG00000184922	1,6532	1,86E-07	0,002	0,358163	1,3872625
PCDHGB2	ENSG00000253910	1,0908	7,48E-05	0,032	0,263124	0,513061
VWF	ENSG00000110799	1,5726	6,38E-05	0,03	0,169839	0,3948225
YPEL1	ENSG00000100027	1,6217	4,62E-05	0,028	0,084572	0,3157415
ENPP7P8	ENSG00000255319	5,2763	9,30E-05	0,032	0,017258	0,2930925
LRRC25	ENSG00000175489	1,9544	9,97E-05	0,033	0,098005	0,257039
MMP23A	ENSG00000215914	4,6844	3,89E-05	0,027	0	0,150381
PTPRE	ENSG00000132334	2,3524	2,08E-05	0,018	0,01637	0,102463
KBTBD11	ENSG00000176595	2,2556	9,82E-06	0,014	0,01583	0,080414
CFAP99	ENSG00000206113	2,9534	0,000107	0,033	0	0,0582065
PRG4	ENSG00000116690	3,1827	9,12E-05	0,032	0	0,022572
MXRA5Y	ENSG00000235649	4,3817	2,07E-05	0,018	0	0,013534

Table S5: List of differentially downregulated mRNAs detected by RNA-seq/DeSeq2

SYMBOL	ENSEMBL ID	log2FC	pvalue	padj	median_tpm_controls	median_tpm_re
MMP1	ENSG00000196611	-2,4295	3,81E-07	0,0016	66,110017	10,527926
HIST1H3B	ENSG00000124693	-1,814	0,00011	0,0336	62,122814	22,0242065
H1-5	ENSG00000184357	-1,7057	0,00015	0,0408	32,791402	13,2317415
HIST1H3G	ENSG00000256018	-2,0331	3,90E-05	0,0269	29,757114	9,709613
HIST1H2AM	ENSG00000233224	-1,1826	0,00017	0,0423	29,698052	14,9299635
HIST1H3C	ENSG00000196532	-1,6045	7,03E-05	0,0313	28,395509	13,4503155
HIST1H2BI	ENSG00000168242	-1,4226	9,29E-05	0,0316	27,530138	12,975357
HIST1H2AJ	ENSG00000182611	-1,9389	4,88E-05	0,0282	23,158921	7,3333775
H3C13	ENSG00000183598	-1,5806	0,0001	0,0333	22,555801	8,0749595
HIST1H3A	ENSG00000198366	-1,0594	0,00017	0,0423	21,689035	14,222951
H2BC11	ENSG00000124635	-1,2224	0,00017	0,0423	17,203942	9,8274835
HIST1H3E	ENSG00000196966	-1,1876	2,44E-05	0,0193	16,254944	8,107657
H3C14	ENSG00000203811	-1,887	8,44E-05	0,0316	14,311764	5,1837525
HIST1H2AL	ENSG00000198374	-1,7036	4,67E-05	0,0282	14,249572	6,398423
HIST1H2BO	ENSG00000196331	-1,4839	0,00012	0,0357	13,766667	8,189454
H2bm	ENSG00000196374	-1,6456	5,97E-05	0,0298	11,986821	5,5537815
H3C12	ENSG00000197153	-1,701	4,84E-05	0,0282	11,036201	4,73655
ANLN	ENSG00000011426	-1,3991	0,00011	0,0333	3,92905	1,904186
HIST1H2BF	ENSG00000197846	-1,4782	0,00013	0,0372	3,632808	1,948649
AURKA	ENSG00000087586	-1,0647	0,0002	0,045	3,347507	1,813594
UBE2T	ENSG00000077152	-1,1739	1,24E-05	0,0145	2,941451	1,824087
CDK1	ENSG00000170312	-1,5402	4,83E-05	0,0282	2,702128	0,705309
CDCA3	ENSG00000111665	-1,6248	8,96E-05	0,0316	2,561554	0,8235845
SLX1B	ENSG00000181625	-2,471	3,24E-07	0,0016	1,991353	0,421757
GINS4	ENSG00000147536	-1,2231	2,57E-05	0,0197	1,562215	0,697043
HJURP	ENSG00000123485	-1,4306	0,0002	0,045	1,508962	0,574157
FBXO5	ENSG00000112029	-1,0189	1,29E-05	0,0145	1,463047	0,952674
POLR3G	ENSG00000113356	-1,162	2,61E-06	0,008	1,218021	0,6074105
CENPU	ENSG00000151725	-1,306	1,80E-05	0,0178	1,018149	0,5498825
SERPINI1	ENSG00000163536	-1,3624	8,65E-05	0,0316	1,009506	0,354877
GINS2	ENSG00000131153	-1,2198	0,00012	0,034	0,988304	0,453577
SPC25	ENSG00000152253	-1,3096	9,15E-05	0,0316	0,892286	0,379472
ADRB2	ENSG00000169252	-1,9189	6,37E-06	0,0097	0,883658	0,4139325
ASF1B	ENSG00000105011	-1,3573	0,00014	0,0385	0,864743	0,550167
CEP97	ENSG00000182504	-1,5051	0,00017	0,0423	0,864455	0,4982575
SLC38A11	ENSG00000169507	-2,417	0,00021	0,0462	0,825463	0,3533545
CDC6	ENSG00000094804	-1,363	8,04E-05	0,0316	0,613035	0,4577705
DTL	ENSG00000143476	-1,7621	4,14E-06	0,0081	0,609068	0,3510095
PITX1	ENSG00000069011	-3,1916	3,37E-06	0,008	0,563843	0,1243665
TRAIP	ENSG00000183763	-1,0316	0,00011	0,0336	0,465813	0,2751235
EVI2A	ENSG00000126860	-1,6478	0,00019	0,0445	0,457765	0,195991
SAPCD2	ENSG00000186193	-1,4823	7,86E-05	0,0316	0,432076	0,1805035
CDKL2	ENSG00000138769	-1,589	5,61E-05	0,0298	0,427906	0,1113425
PLK4	ENSG00000142731	-1,3013	0,00019	0,0448	0,373477	0,20909
AC090204.1	ENSG00000247134	-3,0144	2,42E-05	0,0193	0,354774	0,0449155
MCM10	ENSG00000065328	-1,3616	8,41E-05	0,0316	0,217989	0,15586
AC105339.2	ENSG00000252690	-1,8017	5,73E-05	0,0298	0,197154	0,1041025
CCNA1	ENSG00000133101	-3,0749	6,77E-05	0,0308	0,192588	0,0157815
FAM111B	ENSG00000189057	-1,602	0,00018	0,0424	0,191279	0,111142
BLM	ENSG00000197299	-1,7439	1,87E-05	0,0178	0,157605	0,051824
CHI3L1	ENSG00000133048	-3,1818	9,02E-05	0,0316	0,143634	0,070284
FLT1	ENSG00000102755	-3,3644	2,26E-07	0,0016	0,11438	0,0277975
NEFH	ENSG00000100285	-2,6992	0,00014	0,0392	0,049066	0,018709
DRD2	ENSG00000149295	-4,1277	0,00011	0,0336	0,045105	0,020232
SPOCK2	ENSG00000107742	-2,9766	6,26E-05	0,0303	0,007153	0,005276

Table S6: List of cell type marker mRNAs detected by RNA-seq.

SYMBOL	ENSEMBL ID	log2FC	pvalue	padj	median_tpm_controls	median_tpm_re	Marker
VIM	ENSG00000026025	0,098986	0,611092	0,985	2825,961172	3135,11234	fibroblast
VWF	ENSG00000110799	1,572639	6,38E-05	0,03	0,169839	0,3948225	endothelial
CDH1	ENSG00000039068	1,268513	0,028974	0,467	0,036273	0,0393555	epithelial
ACTN3	ENSG00000248746	-1,50107	0,029073	0,467	0,009346	0,0074165	muscle

The following pages contain the RNA-seq MultiQC report:

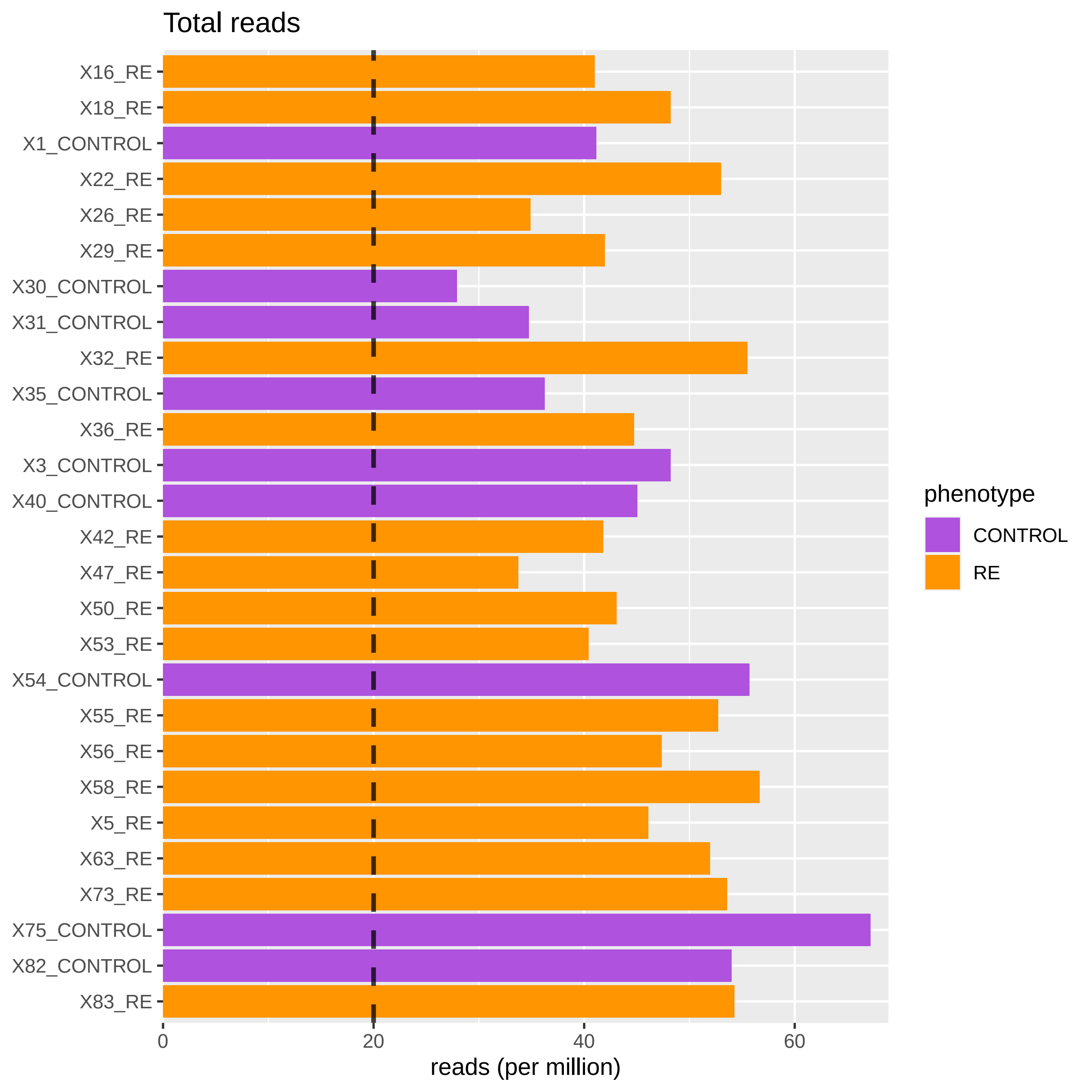

Total reads
X16_RE
X18_RE
X1_CONTROL
X22_RE
X26_RE
X29_RE
X30_CONTROL
X31_CONTROL
X32_RE
X35_CONTROL
X36_RE
X3_CONTROL
X40_CONTROL
X42_RE
X47_RE
X50_RE
X53_RE
X54_CONTROL
X55_RE
X56_RE
X58_RE
X5_RE
X63_RE
X73_RE
X75_CONTROL
X82_CONTROL
X83_RE
0
20
40
60
reads (per million)
phenotype
CONTROL
RE

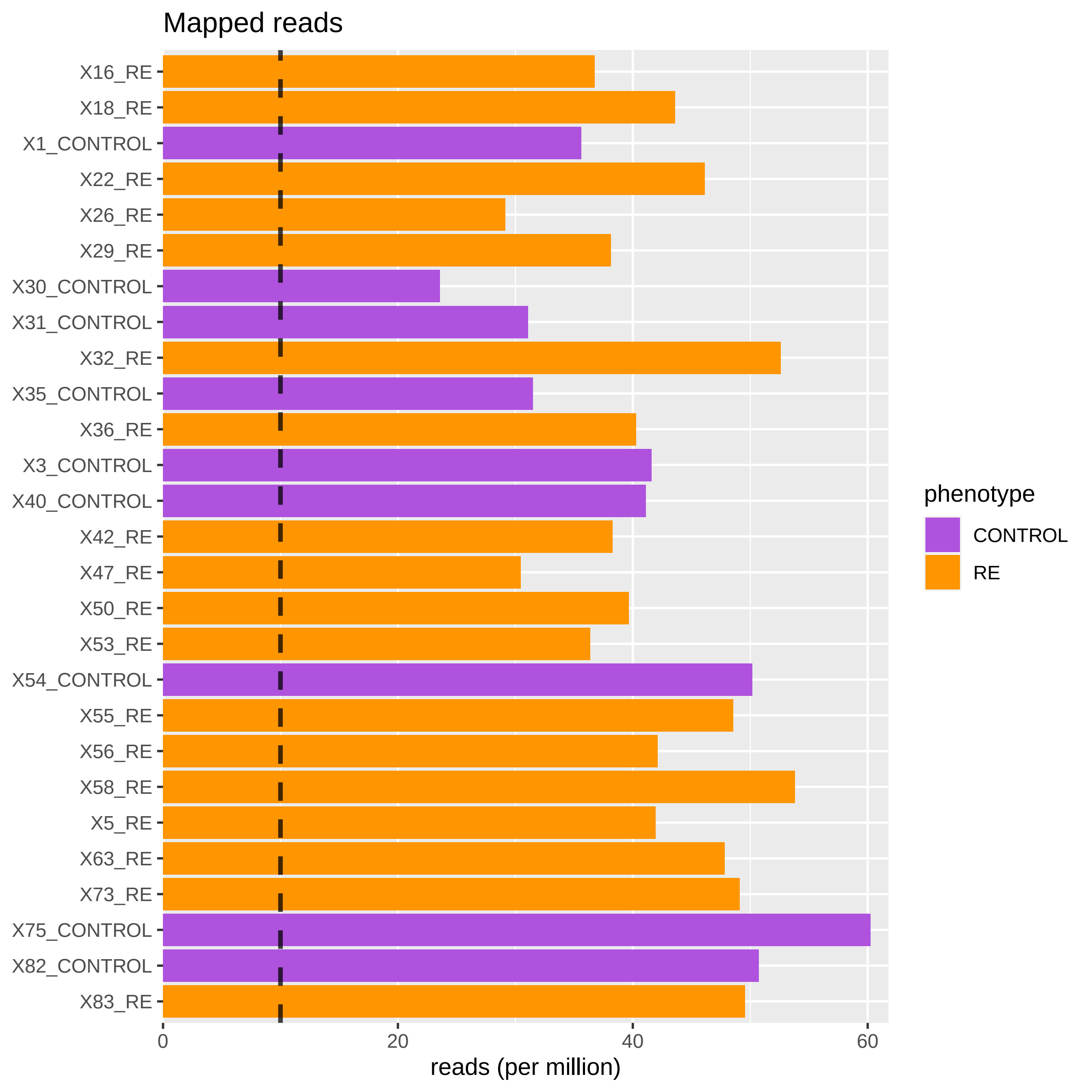

Mapped reads
X16_RE
X18_RE
X1_CONTROL
X22_RE
X26_RE
X29_RE
X30_CONTROL
X31_CONTROL
X32_RE
X35_CONTROL
X36_RE
X3_CONTROL
X40_CONTROL
X42_RE
X47_RE
X50_RE
X53_RE
X54_CONTROL
X55_RE
X56_RE
X58_RE
X5_RE
X63_RE
X73_RE
X75_CONTROL
X82_CONTROL
X83_RE
0
20
40
60
reads (per million)
phenotype
CONTROL
RE

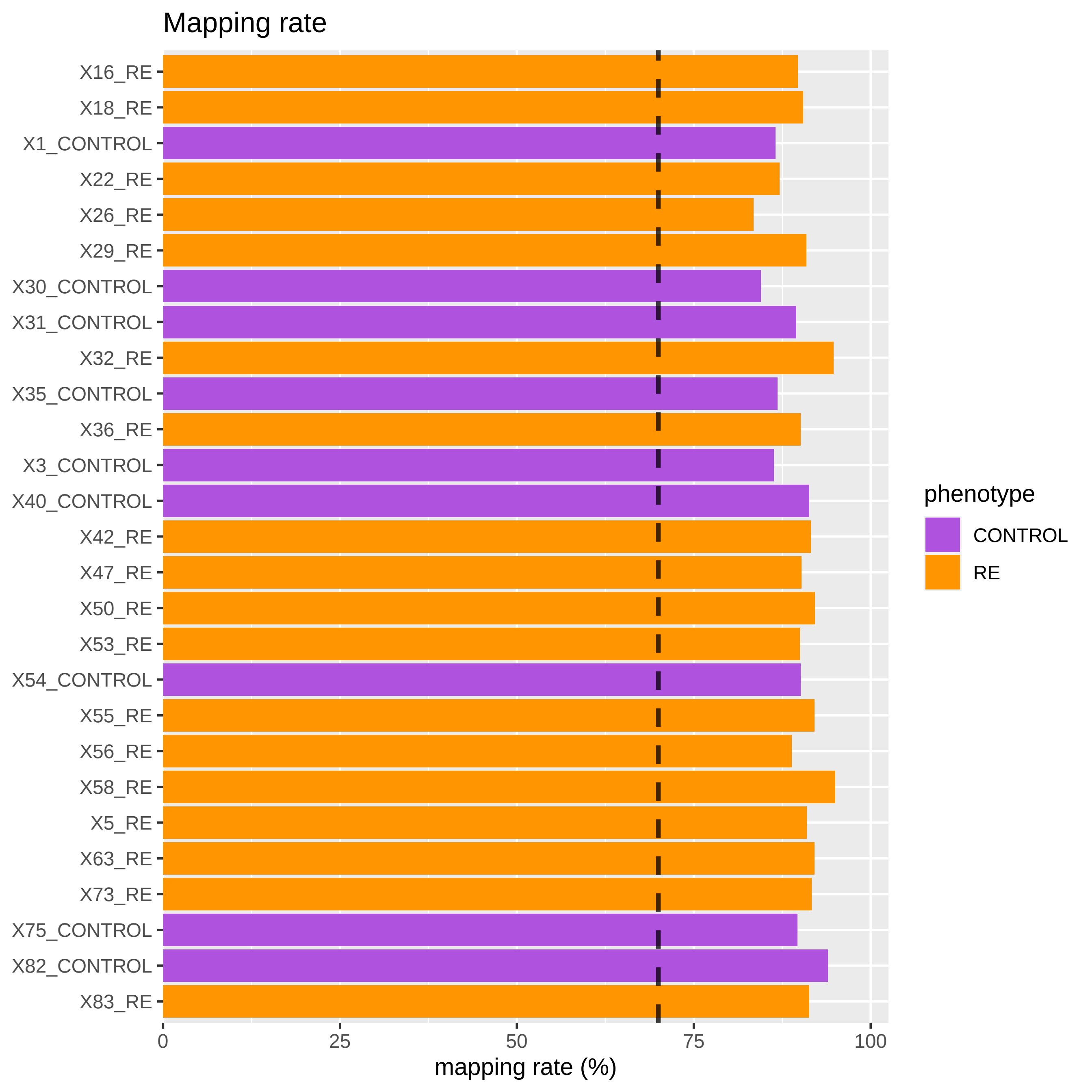

Mapping rate
X16_RE
X18_RE
X1_CONTROL
X22_RE
X26_RE
X29_RE
X30_CONTROL
X31_CONTROL
X32_RE
X35_CONTROL
X36_RE
X3_CONTROL
X40_CONTROL
X42_RE
X47_RE
X50_RE
X53_RE
X54_CONTROL
X55_RE
X56_RE
X58_RE
X5_RE
X63_RE
X73_RE
X75_CONTROL
X82_CONTROL
X83_RE
0
25
50
75
100
mapping rate (%)
phenotype
CONTROL
RE

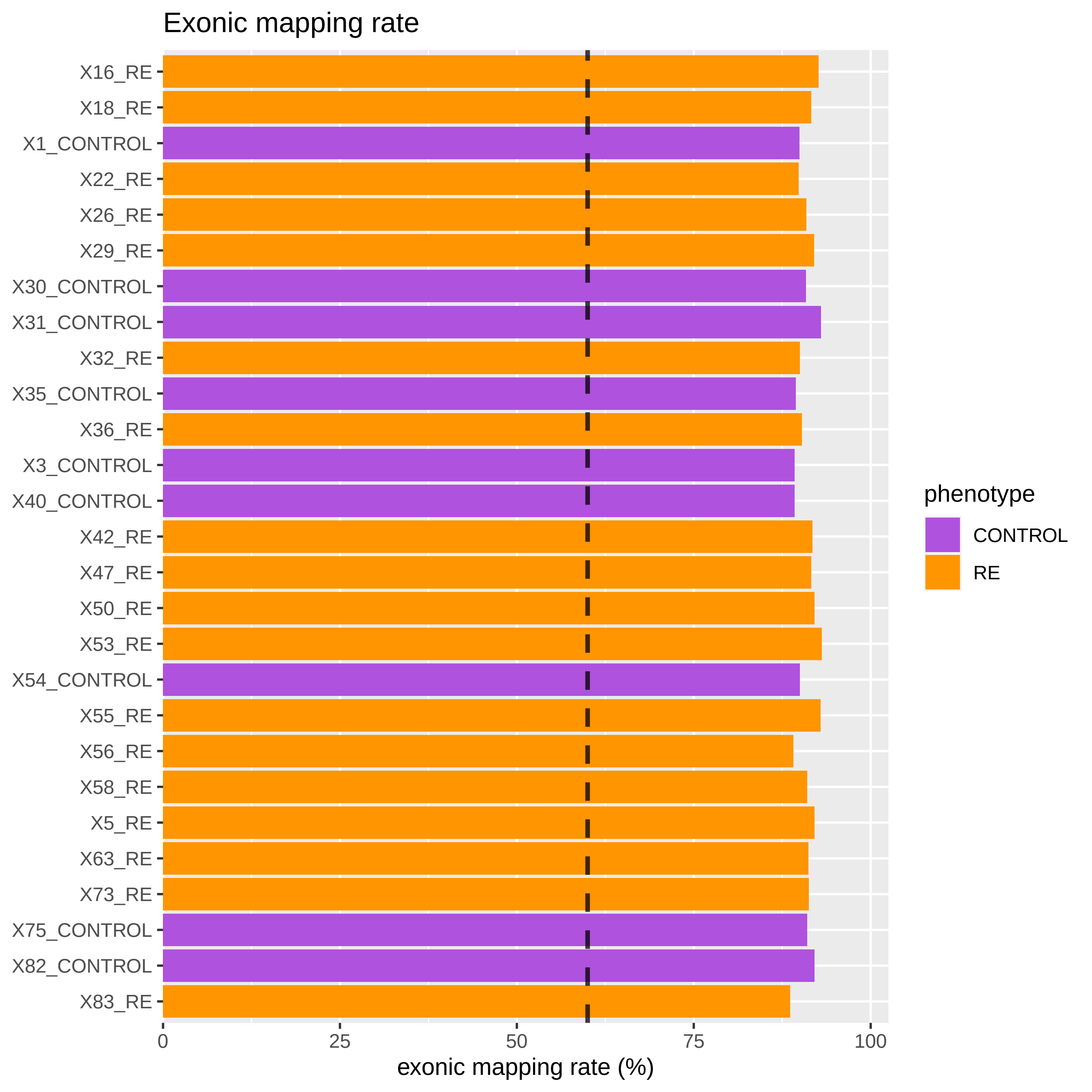
Exonic mapping rate
X16_RE
X18_RE
X1_CONTROL
X22_RE
X26_RE
X29_RE
X30_CONTROL
X31_CONTROL
X32_RE
X35_CONTROL
X36_RE
X3_CONTROL
X40_CONTROL
X42_RE
X47_RE
X50_RE
X53_RE
X54_CONTROL
X55_RE
X56_RE
X58_RE
X5_RE
X63_RE
X73_RE
X75_CONTROL
X82_CONTROL
X83_RE
0
25
50
75
100
exonic mapping rate (%)
phenotype
CONTROL
RE

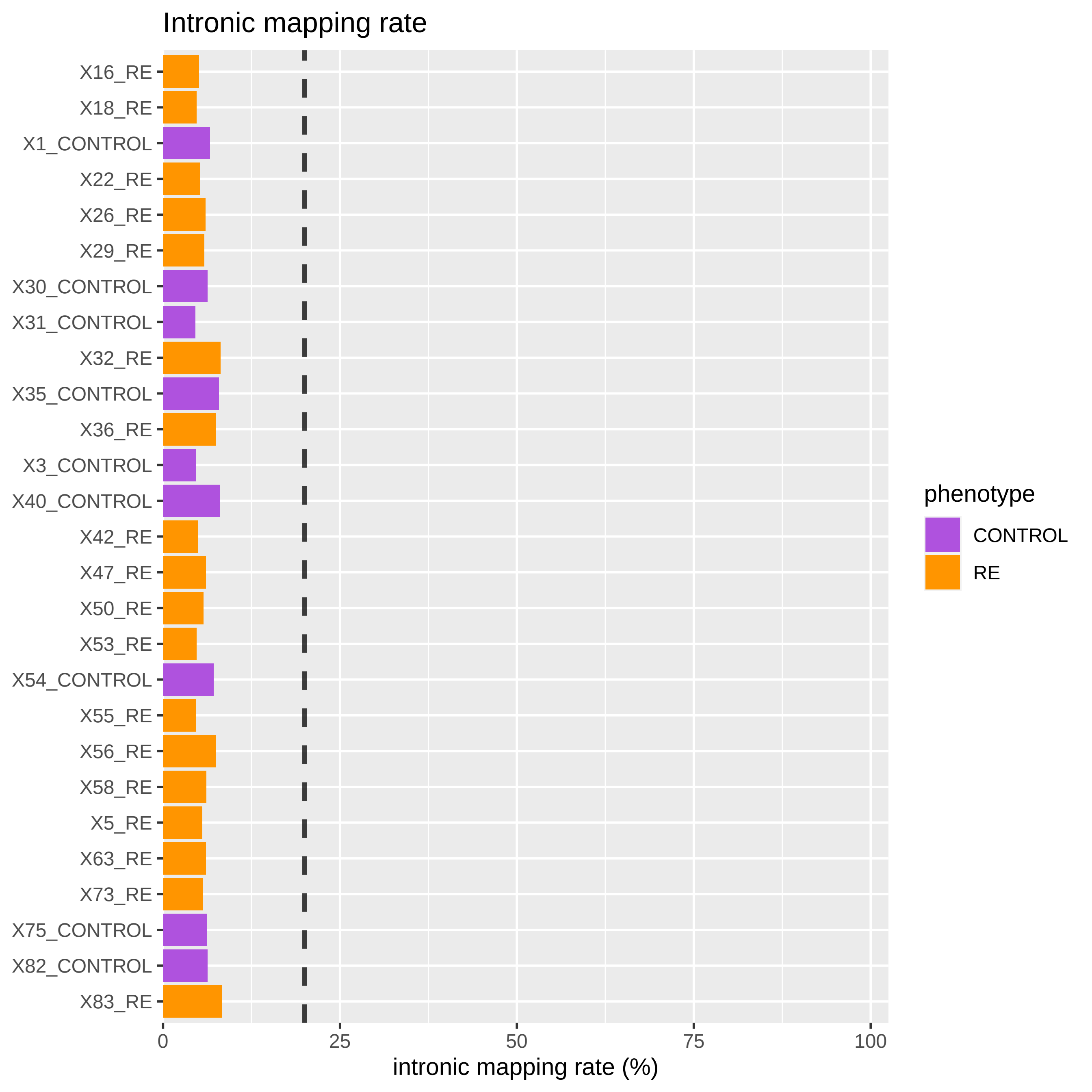
Intronic mapping rate
X16_RE
X18_RE
X1_CONTROL
X22_RE
X26_RE
X29_RE
X30_CONTROL
X31_CONTROL
X32_RE
X35_CONTROL
X36_RE
X3_CONTROL
X40_CONTROL
X42_RE
X47_RE
X50_RE
X53_RE
X54_CONTROL
X55_RE
X56_RE
X58_RE
X5_RE
X63_RE
X73_RE
X75_CONTROL
X82_CONTROL
X83_RE
0
25
50
75
100
intronic mapping rate (%)
phenotype
CONTROL
RE

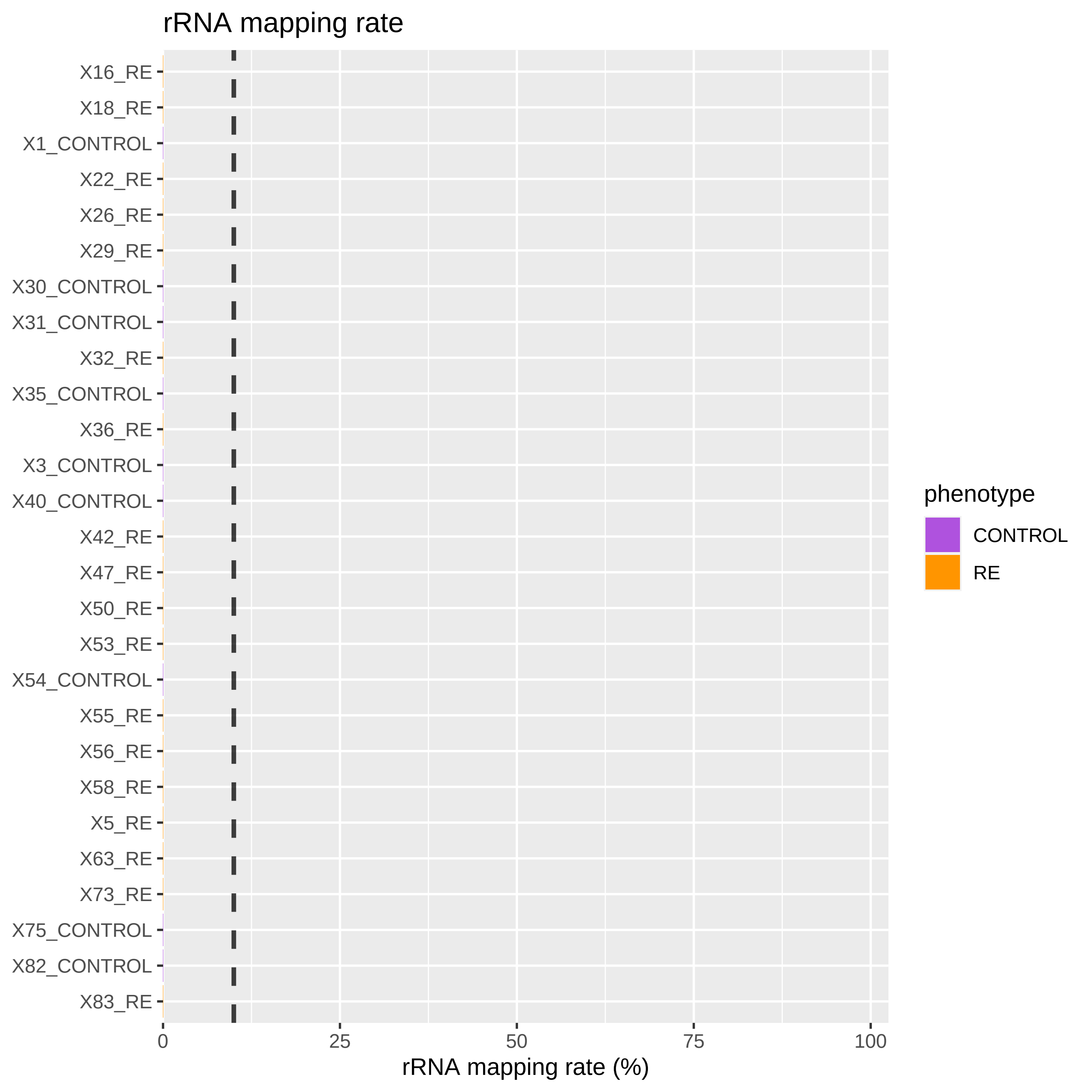
rRNA mapping rate
X16_RE
X18_RE
X1_CONTROL
X22_RE
X26_RE
X29_RE
X30_CONTROL
X31_CONTROL
X32_RE
X35_CONTROL
X36_RE
X3_CONTROL
X40_CONTROL
X42_RE
X47_RE
X50_RE
X53_RE
X54_CONTROL
X55_RE
X56_RE
X58_RE
X5_RE
X63_RE
X73_RE
X75_CONTROL
X82_CONTROL
X83_RE
0
25
50
75
100
rRNA mapping rate (%)
phenotype
CONTROL
RE

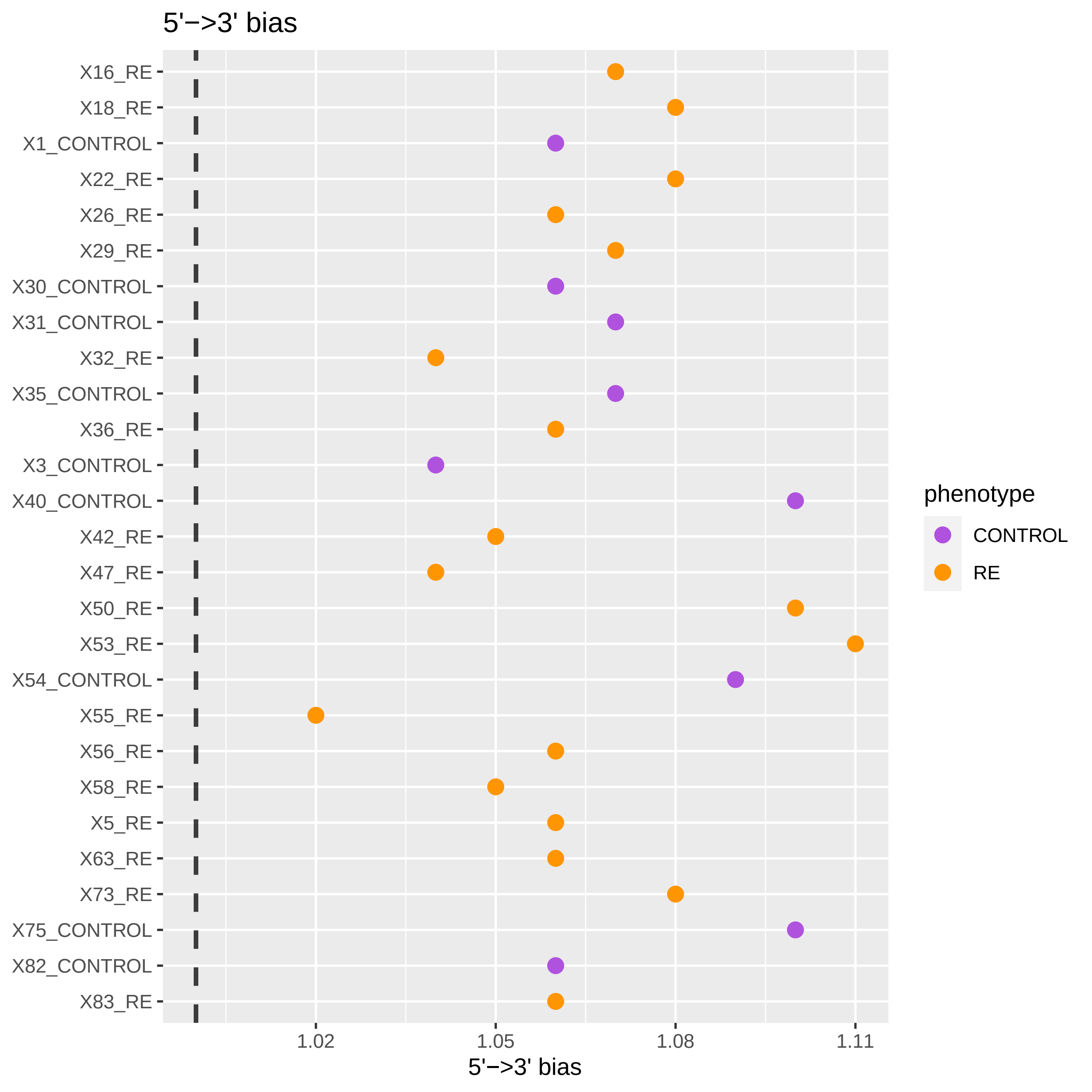

5'−>3' bias
X16_RE
X18_RE
X1_CONTROL
X22_RE
X26_RE
X29_RE
X30_CONTROL
X31_CONTROL
X32_RE
X35_CONTROL
X36_RE
X3_CONTROL
X40_CONTROL
X42_RE
X47_RE
X50_RE
X53_RE
X54_CONTROL
X55_RE
X56_RE
X58_RE
X5_RE
X63_RE
X73_RE
X75_CONTROL
X82_CONTROL
X83_RE
1.02
1.05
1.08
1.11
5'−>3' bias
phenotype
CONTROL
RE

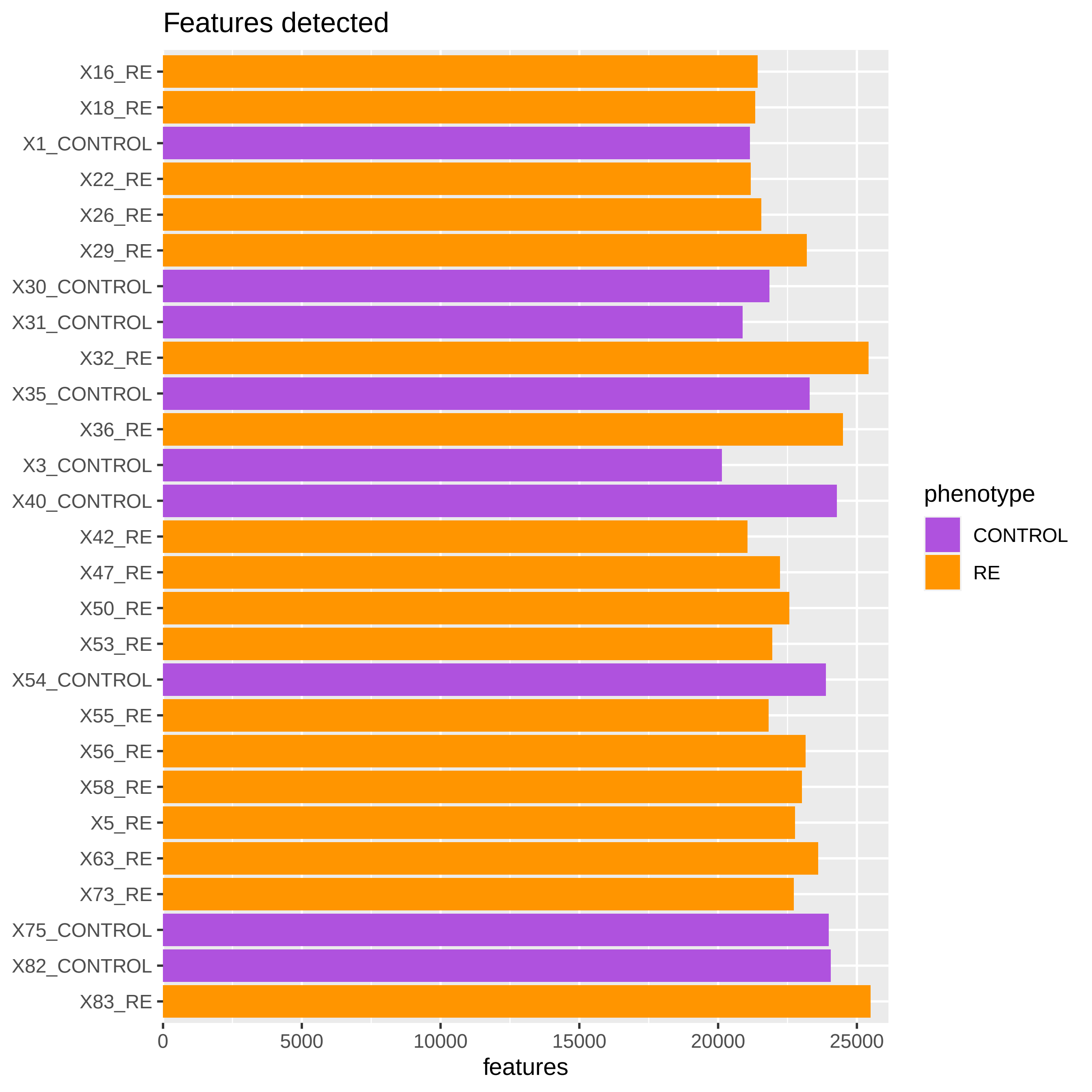

Features detected
X16_RE
X18_RE
X1_CONTROL
X22_RE
X26_RE
X29_RE
X30_CONTROL
X31_CONTROL
X32_RE
X35_CONTROL
X36_RE
X3_CONTROL
X40_CONTROL
X42_RE
X47_RE
X50_RE
X53_RE
X54_CONTROL
X55_RE
X56_RE
X58_RE
X5_RE
X63_RE
X73_RE
X75_CONTROL
X82_CONTROL
X83_RE
0
5000
10000
15000
20000
25000
features
phenotype
CONTROL
RE

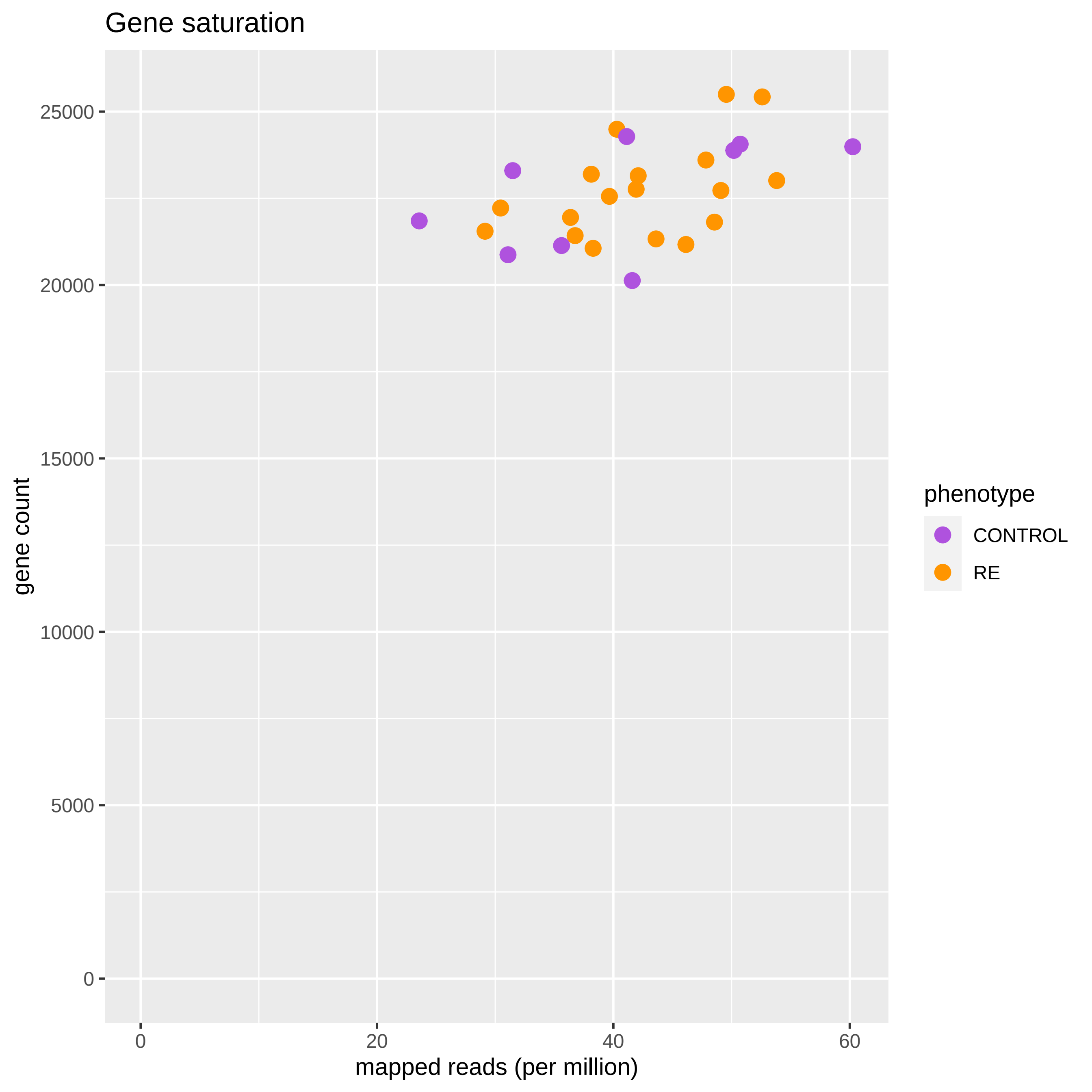

Gene saturation
gene count
25000
20000
15000
10000
5000
0
0
20
40
60
mapped reads (per million)
phenotype
CONTROL
RE

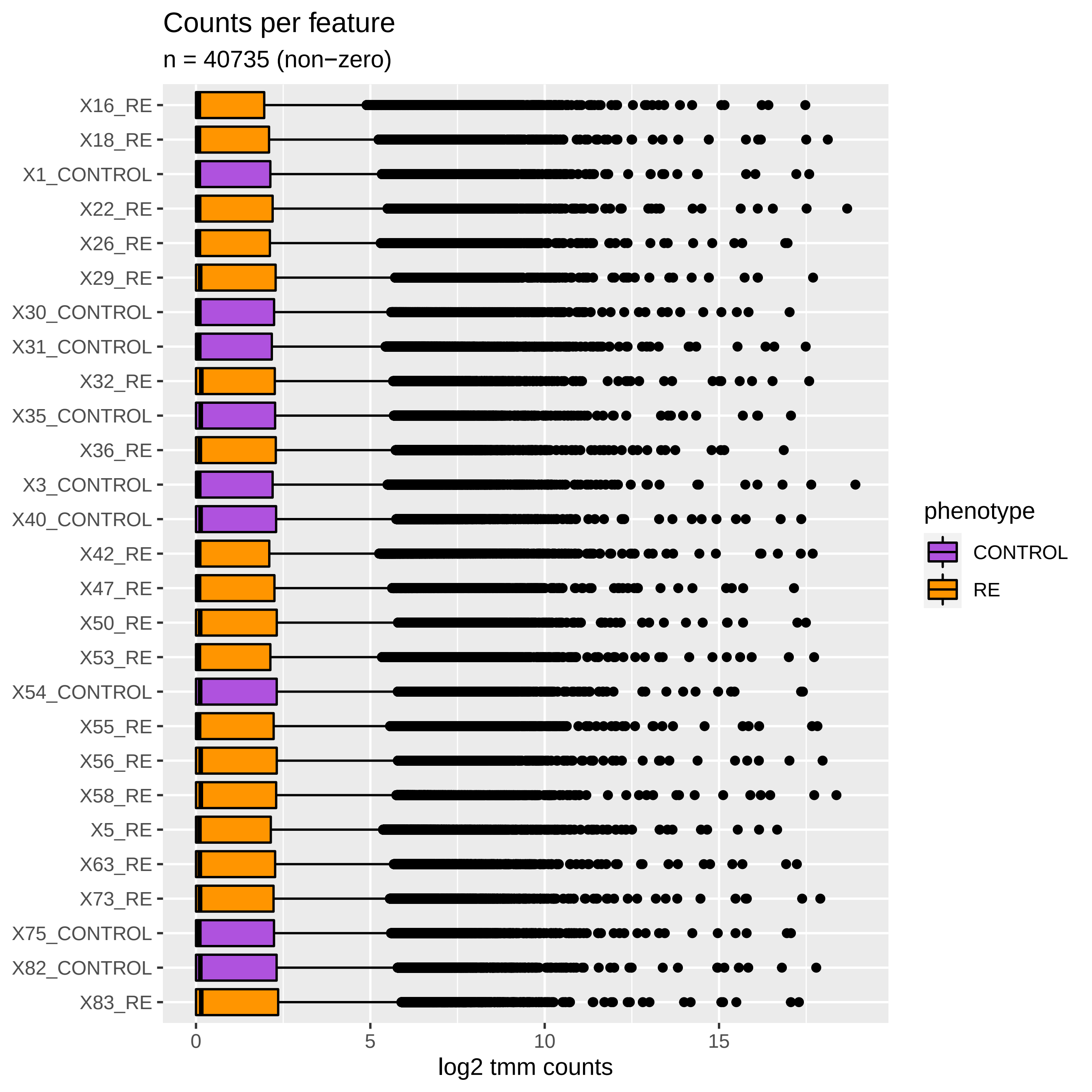

Counts per feature
n = 40735 (non−zero)
X16_RE
X18_RE
X1_CONTROL
X22_RE
X26_RE
X29_RE
X30_CONTROL
X31_CONTROL
X32_RE
X35_CONTROL
X36_RE
X3_CONTROL
X40_CONTROL
X42_RE
X47_RE
X50_RE
X53_RE
X54_CONTROL
X55_RE
X56_RE
X58_RE
X5_RE
X63_RE
X73_RE
X75_CONTROL
X82_CONTROL
X83_RE
0
5
10
15
log2 tmm counts
phenotype
CONTROL
RE

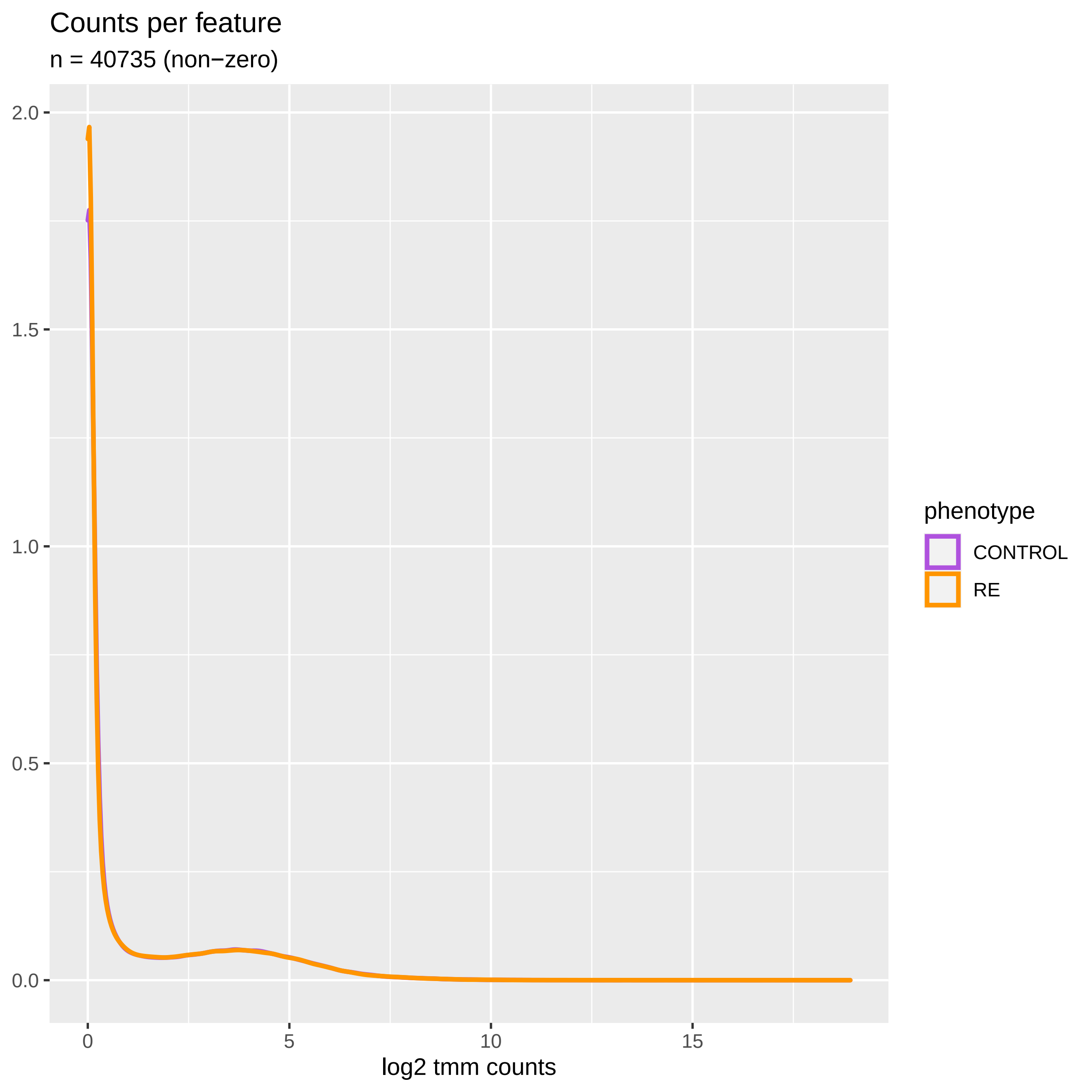
Counts per feature
n = 40735 (non−zero)
2.0
1.5
1.0
0.5
0.0
0
5
10
15
log2 tmm counts
phenotype
CONTROL
RE

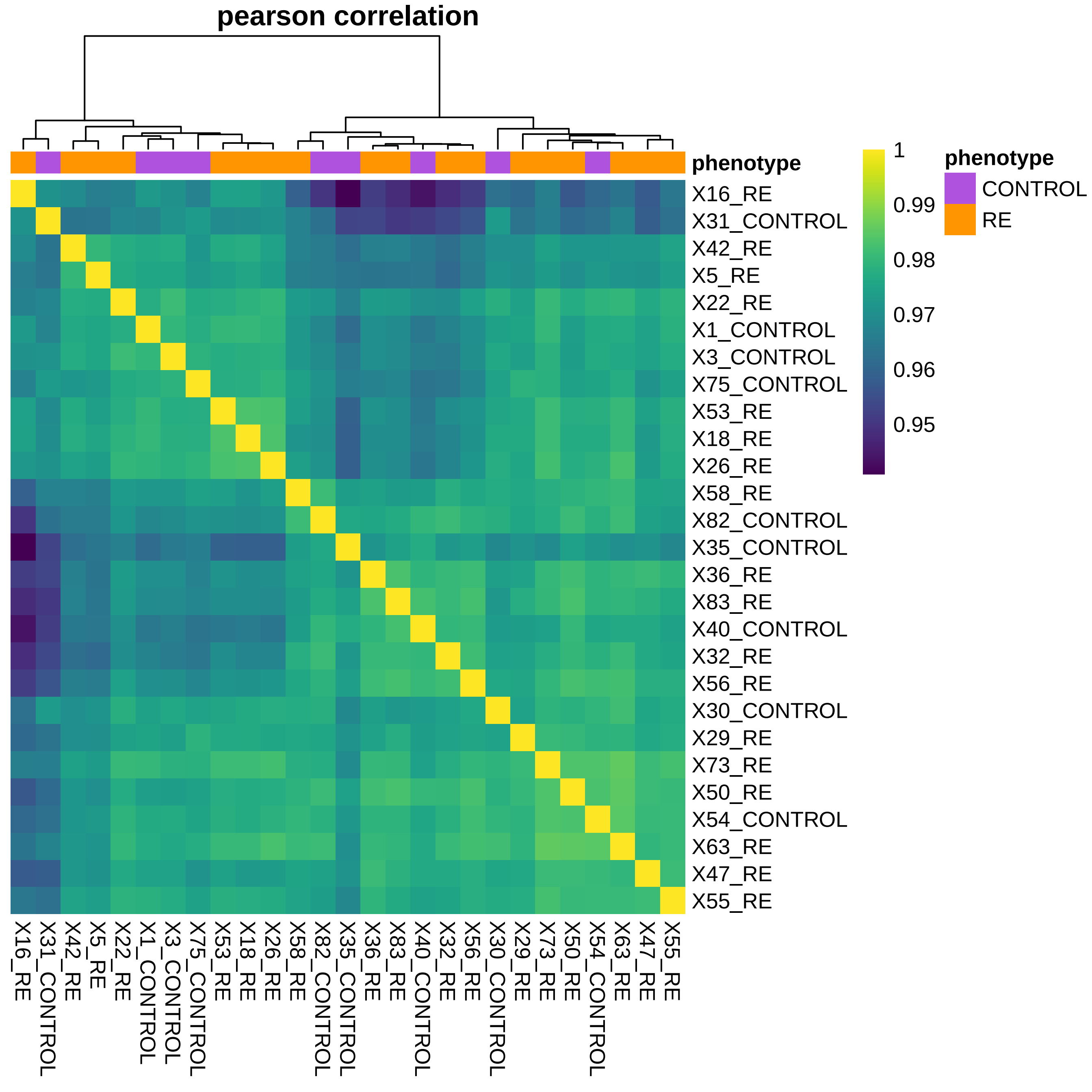
pearson correlation
phenotype
1
0.99
0.98
0.97
0.96
0.95
phenotype
CONTROL
RE
X16_RE
X31_CONTROL
X42_RE
X5_RE
X22_RE
X1_CONTROL
X3_CONTROL
X75_CONTROL
X53_RE
X18_RE
X26_RE
X58_RE
X82_CONTROL
X35_CONTROL
X36_RE
X83_RE
X40_CONTROL
X32_RE
X56_RE
X30_CONTROL
X29_RE
X73_RE
X50_RE
X54_CONTROL
X63_RE
X47_RE
X55_RE

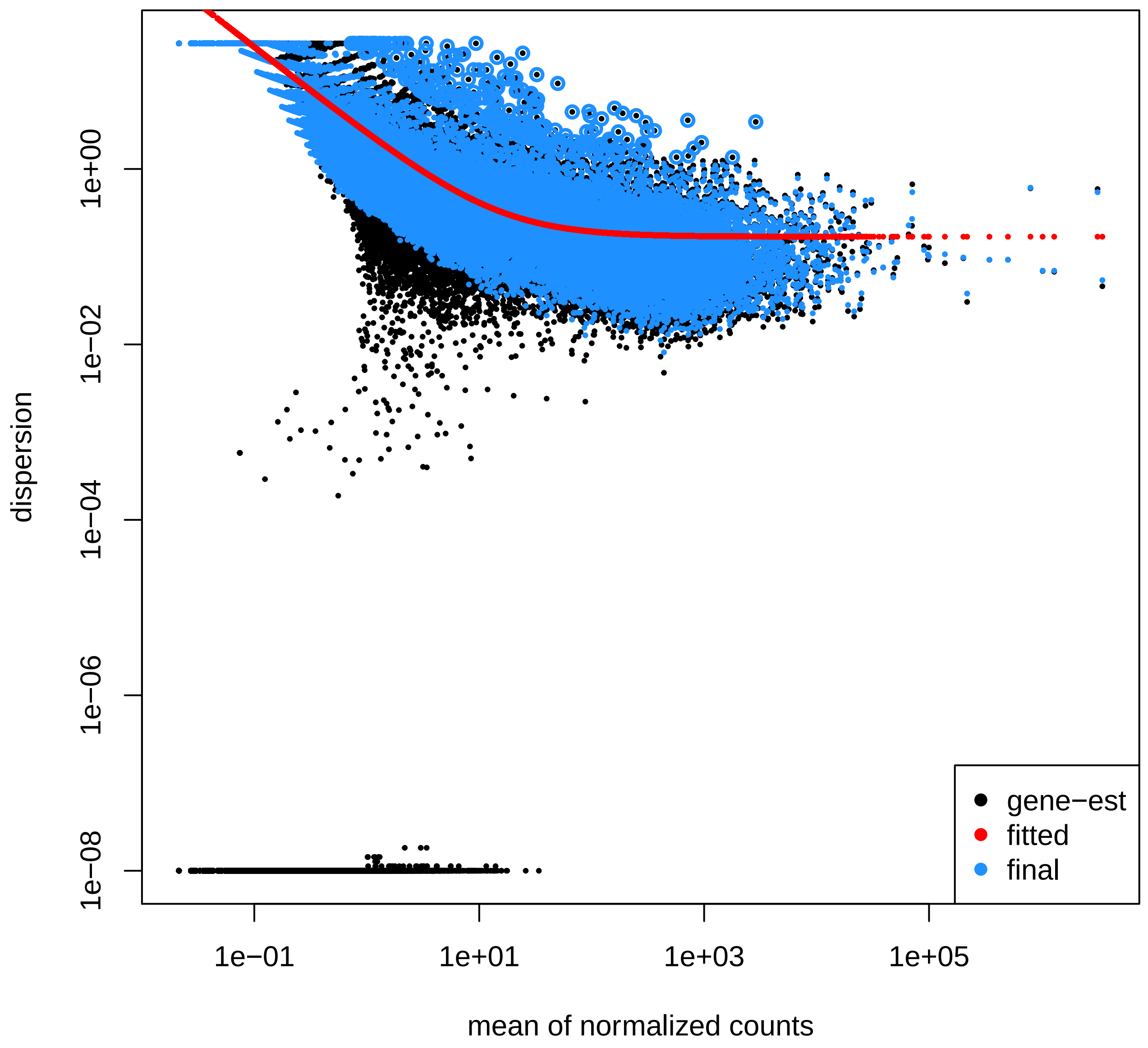
dispersion
1e+00
1e−02
1e−04
1e−06
1e−08
1e−01
1e+01
1e+03
1e+05
mean of normalized counts
gene−est
fitted
final
